# High Power Factor vs. High *zT*—A Review of Thermoelectric Materials for High-Temperature Application

**DOI:** 10.3390/e21111058

**Published:** 2019-10-29

**Authors:** Mario Wolf, Richard Hinterding, Armin Feldhoff

**Affiliations:** Institute of Physical Chemistry and Electrochemistry, Leibniz University Hannover, Callinstraße 3A, D-30167 Hannover, Germany

**Keywords:** thermoelectric materials, energy harvesting, energy materials

## Abstract

Energy harvesting with thermoelectric materials has been investigated with increasing attention over recent decades. However, the vast number of various material classes makes it difficult to maintain an overview of the best candidates. Thus, we revitalize Ioffe plots as a useful tool for making the thermoelectric properties of a material obvious and easily comparable. These plots enable us to consider not only the efficiency of the material by the figure of merit *zT* but also the power factor and entropy conductivity as separate parameters. This is especially important for high-temperature applications, where a critical look at the impact of the power factor and thermal conductivity is mandatory. Thus, this review focuses on material classes for high-temperature applications and emphasizes the best candidates within the material classes of oxides, oxyselenides, Zintl phases, half-Heusler compounds, and SiGe alloys. An overall comparison between these material classes with respect to either a high efficiency or a high power output is discussed.

## 1. Introduction

At a time when raw fossil materials are becoming scarcer and the demand for regenerative energies is relentlessly rising, the use of energy harvesting systems has gained an ever-increasing interest [1]. Regardless of whether it is from industrial processes, mechanical processes, or the transportation sector, the amount of wasted energy currently remains enormous. In 2017, the estimated energy consumption in the U.S. was shown to be approximately 67% wasted energy [2]. At this point, energy harvesting comes into play, converting even small amounts of wasted energy in the form of heat, light, vibration, or movement into usable energy [3]. Since most of this wasted energy is in the form of heat, the conversion of thermal energy to electrical energy via thermoelectric generators is an attractive solution. The associated energy conversion is based on the thermoelectric effect, which is the simplest way for direct energy conversion from dissipated heat into electrical energy.

Discovered by T.J. Seebeck in 1821, the first thermoelectric effect (Seebeck effect) describes the direct conversion of thermal energy into electrical energy, which Seebeck demonstrated by thermally inducing an electrical current by heating two different electrical conductors. Together with the Peltier effect (1834), which describes the heating or cooling effect of an electrical current in a thermocouple, and the work of W. Thomson on the thermoelectric effect in homogeneous conductors (Thomson effect), the basis of thermoelectricity was laid [4]. In the first half of the 20th century, the term ‘figure of merit’ was introduced, and the first theoretical approaches were made in designing a material with a high energy conversion efficiency. In 1957, A.F. Ioffe defined the figure of merit *zT* as a function of the electrical conductivity, the Seebeck coefficient, and the thermal conductivity of the material [5]. However, thermoelectric energy conversion has been too inefficient for most applications for a long time. Theoretical descriptions of nanostructural engineering and superlattice structures paved the way to significantly improved *zT* values, which strongly increased research on thermoelectric materials in the mid 1990s [6]. Today, the improvement and development of thermoelectric materials still have the goals of gaining higher efficiencies and power outputs. Within this research field, various materials from wide-ranging material classes, such as metallics and intermetallics [7,8], oxide-based ceramics [9,10,11], chalcogenide compounds [12], and polymers [13] have been investigated. It is important to compare the efficiencies and resulting power outputs of these materials to draw the correct conclusions when actual generators for applications are manufactured. The purpose of this review is to convey descriptive comparisons, which is realized by two different types of Ioffe plots. These plots allow a direct comparison of the thermoelectric properties of different materials, which is vital for prospective research [14].

### 1.1. Thermoelectric Parameters

Discussing thermoelectricity requires an understanding of some fundamental parameters, which are briefly described in the following. Thermoelectric energy conversion is based on local coupling of fluxes of charge carriers and entropy. When a thermoelectric material is simultaneously exposed to local gradients of temperature (i.e., ∇*T*) and an electrochemical potential of charge carriers (i.e., $\nabla \frac{\tilde{\mu }}{q}$) the local flux densities of charge *j*_q_ and entropy *j*_s_ are given by the following transport equation [15]:

(1) $$\left(\begin{array}{c} j_{q}\\ j_{s} \end{array}\right)=\left(\begin{array}{cc} \sigma & \sigma \cdot \alpha \\ \sigma \cdot \alpha & \sigma \cdot \alpha^{2}+\Lambda \end{array}\right)\cdot \left(\begin{array}{c} -\nabla \frac{\tilde{\mu }}{q}\\ -\nabla T \end{array}\right).$$

The thermoelectric material tensor, which appears here, is characterized by three material parameters: the isothermal electrical conductivity σ, the Seebeck coefficient α, and the electrically open-circuited entropy conductivity Λ. The latter is linearly related to the traditionally used heat conductivity λ via the absolute temperature *T* as described in Equation (2) [15,16,17]. In the context of this review, thermal conductivity is a generic term, that covers both entropy conductivity and heat conductivity. Here, it is advantageous to address the thermal conductivity by the more fundamental entropy conductivity, as we will see when comparing materials.

Considering entropy as a central primitive quantity of equal rank to electric charge comes with the benefit of an easy understanding of the physics of thermoelectricity. A local coupling of the fluxes of these substance-like quantities is described by Equation (1) [15]; this is in contrast to the cumbersome traditional approach, which introduces generalized forces and a kinetic matrix [18] instead of thermodynamic potential gradients and a material tensor. Naturally, for each substance-like quantity, a conductivity is assigned to the material. The material tensor is symmetric by principle, and an elaborate discussion of the reciprocity of Onsager coefficients is superfluous [15]. A further advantage is that the currents of thermal energy (heat) and electrical energy (or electrochemical energy), which accompany the fluxes of entropy and charge, can be treated separately. The entropy and charge fluxes allow us to consider energy conversion and its efficiency in a thermoelectric material apart from the device. When expressed with the entropy conductivity, the figure of merit *zT* in Equation (2) is purely a material parameter that depends only implicitly on temperature. Moreover, the appearance of two substance-like quantities in Equation (1) to be transported through a thermoelectric material allows for an integration of thermoelectricity into a broad picture of coupled transport processes (e.g., diffusion, viscous flow, entropy conduction and electric conduction) and benefits from analogies. A reader who is interested in more details is referred to the discussion about the properties of heat by Fuchs [19] and the comparative overview by Job and Rüffler [17].

The tensor element *M*_22_ in Equation (1) directly leads to parameters that describe the performance of a thermoelectric material. The most commonly used parameter is the dimensionless figure of merit *zT*, which describes the relation of the power factor σ$\alpha^{2}$ and the entropy conductivity Λ in Equation (2).

(2) $$zT=\frac{\sigma \cdot \alpha^{2}}{\Lambda }=\frac{\sigma \cdot \alpha^{2}}{\lambda }\cdot T.$$

The figure of merit *zT* indicates the maximum achievable power conversion efficiency of a thermoelectric material [16]:

(3) $$\eta_{\max}=\frac{\sqrt{1+\mathrm{zT}}-1}{\sqrt{1+\mathrm{zT}}+1}\cdot \eta_{\mathrm{Carnot}}.$$

In contrast, the power factor σ$\alpha^{2}$ is proportional to the maximum achievable power output of a material and the temperature difference ∆*T* [20]:

(4) $$P_{\mathrm{el},\max}\propto \sigma \alpha^{2}\cdot (∆T{)}^{2}.$$

**Figure 1:**
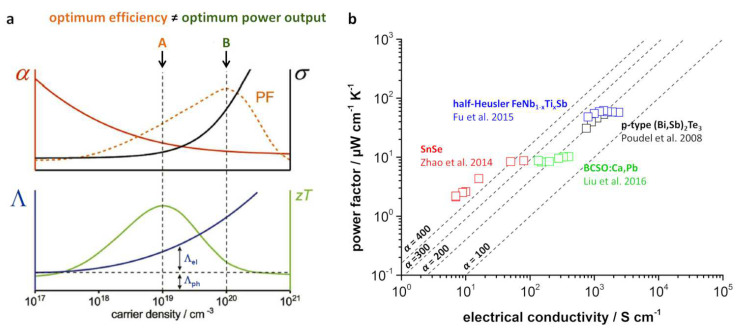
(**a**) Seebeck coefficient α, electrical conductivity σ, entropy conductivity Λ, and the resulting power factor σ$\alpha^{2}$ and *zT* value as a function of the charge carrier density; adapted from [20] with permission from Elsevier; (**b**) Type-I Ioffe plot of various reported high-*zT* materials [11,12,21,22]. Seebeck coefficient α is given in μV K^−1^. Different data points for the same material refer to different temperatures.

For some applications, the power factor may have the same relevance as the efficiency described by the *zT* value. As shown in Figure 1a, for a thermoelectric material, the optimum efficiency and optimum power output as a function of the carrier density differ, leading to a possible optimization of one parameter for the desired properties or a special application.

Narducci [23] also emphasized that the figure of merit *zT* may be an inappropriate parameter to rate and compare different materials for some applications. Especially for applications at high temperature, the power factor $\sigma \alpha^{2}$ could be a better quality criterion. In this review, rather than just comparing the figure of merit *zT*, we will also look at the power output and the entropy conductivity of different materials by comparing them via Ioffe plots. We distinguish between a type-I Ioffe plot, which considers the power factor σ$\alpha^{2}$ as a function of the electrical conductivity σ, and a type-II Ioffe plot, which considers the entropy conductivity Λ as a function of the electrical conductivity σ. Figure 1b displays a type-I Ioffe plot of various reported materials from different classes with high figure of merit *zT* values. Although all of the shown materials exhibit exceptionally high *zT* values in their specific material class, the electrical conductivity σ as well as the power factor vary over several orders of magnitude. Furthermore, type-II Ioffe plots will be vital for an in-depth comparison of different thermoelectric materials later on. Since the entropy conductivity can be displayed in the same unit and scale as the power factor, the type-II Ioffe plot enables an easy comparison of electrical and thermal properties and observable *zT* values.

### 1.2. Doping and Band Structure Engineering

A prevailing challenge when facing the improvement of a thermoelectric material is coupling all three relevant material parameters with each other, which can be managed to a certain degree. The most common approach to enhancing the performance of thermoelectric materials is doping, which can have a huge impact on the electrical conductivity, Seebeck coefficient, and thermal conductivity. Several parameters, such as carrier density, mobility, effective mass, and the band structure, are effectively influenced by proper doping.

Figure 1a shows an optimum carrier density for a balanced electrical conductivity and Seebeck coefficient at a given temperature, which varies between an optimum power factor and *zT* value. This means that adjusting the carrier density via doping is highly advised for either high power or high efficiency applications [23]. The dopant may influence the carrier density of a material by introducing defect-enabled mechanisms including point defects [1]. However, the optimum carrier density is temperature-dependent and increases approximately with $T^{3/2}$ [5]. Thus, either further material engineering is required or the carrier density needs to be optimized for a specific temperature range according to the application field.

Generally, the Seebeck coefficient decreases with increasing carrier concentration, as shown in Figure 1, and the electrical conductivity is oppositely coupled. However, the doping-induced convergence of valence or conduction bands to increased valley degeneracy has proven to increase the electrical conductivity while maintaining the Seebeck coefficient [24]. This is explained by the Seebeck coefficient being dominated by the smaller value of several bands. If degeneracy of several bands is achieved, the Seebeck coefficient is stable, but the electrical conductivity substantially rises [24,25]. Therefore, band convergence is an effective way to improve the power factor of a material.

Additionally, doping strongly influences the thermal properties of the material. The thermal conductivity expressed as entropy conductivity Λ was introduced in the tensor element *M*_22_ in Equation (1). Conventionally, the thermal conductivity is divided into a phonon contribution and an electronic contribution [5]. Influencing the phonon contribution to the thermal conductivity via scattering mechanisms is well investigated and can be divided into size-relevant dimensions. The atomic scale is influenced by single doping or cross substitution, which results in point defects within the crystal lattice. These defects effectively delay the phonon propagation by scattering when the lattice mismatch between the host and the dopant is sufficient and the mass difference as well as the dopant amount are high [26]. The next relevant dimension for scattering phonons is the nanoscale, which leads to so-called nanostructuring [27]. Nanoscaled defects can be introduced in-situ by forcing the precipitation of a second phase [28] or ex-situ by mixing the nanoscaled second phase with a host phase [29]. However, the phonon propagation within the second phase is rather unpredictable, making it difficult to design a proper system. Temperature stability is another issue since elevated temperatures may change the scale of the precipitates and diminish the achieved effect [27]. The main part of the thermal energy is propagated by short- and medium-wavelength phonons, which are effectively hindered by point defects and nanostructuring. The residual long-wavelength phonons can be influenced by defects in the range of a few micrometers or submicrometers, namely, the mesoscale. This effect is typically active for polycrystalline materials with mesoscale grains [30]. Additional phonon scattering may appear at heteromaterial interfaces of composite systems [31]. However, the electronic transport may be reduced by these grains, making a consideration of maximum power output vs. efficiency necessary. If the scattering of the phonons is achieved at all three length scales, the term all-scale hierarchical architecture is used [32]. Only a few studies achieve all-scale phonon scattering, but when regarding the overall reduction of the phonon contribution to the thermal conductivity, decreases of more than 50% are possible [33].

While the phonon contribution to the thermal conductivity has been widely studied, the electronic contribution is not straightforward. For degenerate semiconductors with charge carriers of the same sign, the commonly used separation into both contributions fits well, and the Wiedemann–Franz relation Λ = *L*_0_ · σ with the Sommerfeld value of the Lorenz number *L*_0_ = 2.4453 · 10^−8^ W Ω/K^2^ [34] can be applied to describe the electronic contribution. However, for nondegenerate semiconductors and especially for materials with charge carriers of both signs, the electronic contribution significantly increases, resulting in a deviation of the Sommerfeld value, as stated by Ioffe [5]. Thus, the relationship may lead to questionable results when applied to all kinds of thermoelectric materials and must be considered carefully. As a result, doping may have a nonnegligible influence on the electronic contribution to the thermal conductivity.

### 1.3. Thermoelectric Materials for High-Temperature Applications

**Figure 2:**
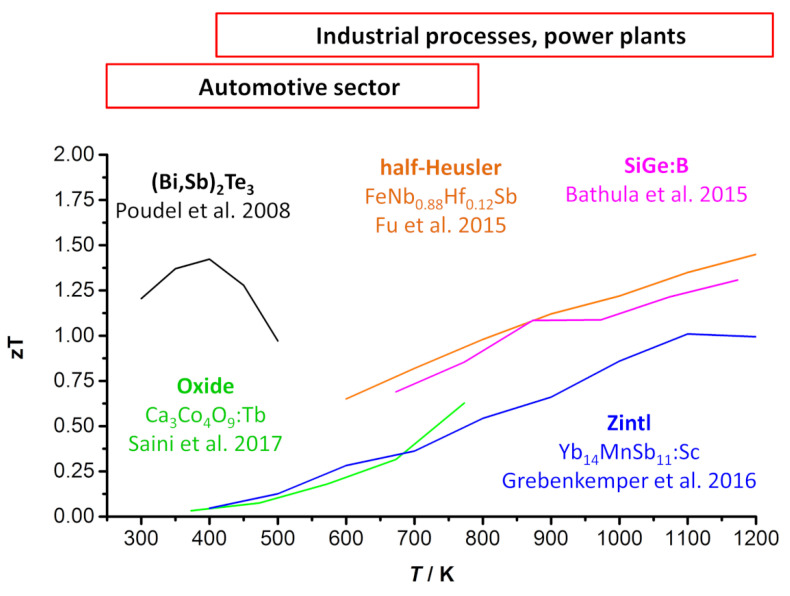
*zT* values as a function of temperature for several thermoelectric material classes [21,22,39,40,41]. While bismuth telluride shows the highest *zT* value at low temperatures, different kinds of materials are interesting for high-temperature applications.

As state-of-the-art materials for thermoelectric power conversion, bismuth telluride (Bi_2_Te_3_) and lead chalogenide PbX (X = S, Se or Te) compounds long exhibited the highest *zT* near room temperature and approximately 600 to 700 K, respectively [35]. However, due to the toxicity of telluride and lead, investigations into less toxic alternatives have attracted strong attention in recent years. Several promising materials or material classes have been reported since and have been discussed in detailed reviews, e.g., copper and tin chalcogenides [35], oxide-based materials [9,36], intermetallic compounds [8,37], and organic polymers [13,38]. Since each material typically has an optimum temperature range for its most efficient thermoelectric power conversion, the materials do not necessarily compete but complement each other with respect to a specific application. Figure 2 shows the *zT* values of several different thermoelectric material classes as a function of temperature. There are multiple promising alternatives for commercially used bismuth telluride at high operating temperatures.

In this review, we take a closer look at these promising materials for high-temperature applications (>700 K), e.g., power plants, industrial processes, and the automobile industry [20,42]. Therefore, oxide-based materials and several intermetallic compounds such as Zintl phases and half-Heusler compounds will be discussed and compared in terms of the power factor and the figure of merit *zT*. In this context, the focus is on the thermoelectric properties at the material level. Please note that the utilization of thermoelectric materials in a device comes alongside additional important tasks such as contact resistivity and the variation in thermoelectric properties in an applied temperature gradient. It has been emphasized that for a thermoelectric device, the average properties, such as the average *zT*, within the respective temperature range are the key parameters instead of the peak properties [43,44,45]. As mentioned, within this review, the discussed thermoelectric compounds will only be evaluated at their respective material level.

## 2. Oxides and Oxyselenides

### 2.1. Thermoelectric Oxides

Oxide-based thermoelectric materials generally exhibit an inferior *zT* compared to that of telluride and selenide compounds, but show a much higher chemical and thermal stability, thus allowing high operating temperatures and large temperature gradients [46]. Consequently, such materials are very promising for high-temperature applications in areas such as the automobile sector or industrial furnaces. Additionally, raw materials of such oxide-based ceramics are less toxic than other materials and therefore easier to process. The first works that predicted good thermoelectric properties in layered crystal structures [47] and the first report of Na_x_CoO_2_ [48] were published in the mid 1990s. Later, manganites and cobaltites, which show strong spin and orbital fluctuations in the d-electron system and a strong Jahn–Teller effect, became the focus of research [9]. Today, oxide-based thermoelectric materials represent a group of materials with good chemical and temperature stability. In general, oxide-based thermoelectric materials exhibit high Seebeck coefficients, but only a medium electrical conductivity and moderate thermal conductivity. By adjusting these parameters via nanostructuring, doping, and defect engineering, these materials can be tuned to reach high *zT* values up to 1. In this context, we will have a close look at *p*-type layered cobaltites (Na_x_CoO_2_, Ca_3_Co_4_O_9_, Bi_2_Ca_2_Co_2_O_9_) and the most common *n*-type oxide-based materials (ZnO, CaMnO_3_, SrTiO_3_). As already mentioned, the focus of this work is on a comparison and evaluation of the power factor and the figure of merit *zT*. For a more detailed discussion of the physical properties of thermoelectric oxides, e.g., for oxide-based materials [9,36,49] or BiCuSeO [49,50,51], the reader is referred to other review articles.

#### 2.1.1. *p*-Type Layered Cobaltites

**Figure 3:**
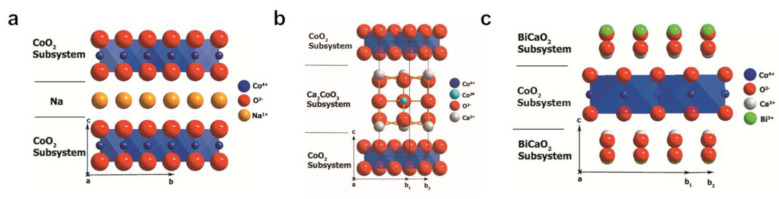
Crystal structures of (**a**) Na_x_CoO_2_, (**b**) Ca_3_Co_4_O_9_ (CCO) and (**c**) Bi_2_Ca_2_Co_2_O_9_. Adapted from [10] with permission from Elsevier.

Since the discovery of Na_x_CoO_2_, several layered cobaltite compounds with analogous structures have been found. In addition to Na_x_CoO_2_, two other promising compounds, namely, Ca_3_Co_4_O_9_ and Bi_2_A_2_Co_2_O_9_ (A = alkaline-earth metal), will be discussed. Figure 3 shows the crystal structures of these compounds. Na_x_CoO_2_ consists of a hexagonal-layered structure with CoO_2_ sheets separated by disordered Na layers. In Ca_3_Co_4_O_9_ and Bi_2_A_2_Co_2_O_9_ (A = alkaline-earth metal), the Na layer is replaced by Ca_2_CoO_3_ or Bi_2_A_2_O_4_ substructures, respectively. In these structures, the CoO_2_ sheets represent an electron-conducting layer, which is described as an ‘electron crystal’, while the salt-like separating layers work as a ‘phonon glass’ and reduce the thermal conductivity of the material, resulting in a high *zT* [1,46].

The thermoelectric parameters of these layered cobaltites are strongly influenced by the exact stoichiometry of the compound. In Na_x_CoO_2_, the amount of Na in the disordered phonon glass layer influences the phonon scattering and the electronic properties [52,53]. In Bi_2_A_2_Co_2_O_9_ (A = alkaline-earth metal), the thermoelectric properties can be influenced by the amount of Co [54]. The most investigated compounds of Bi_2_A_2_Co_2_O_9_ have A = Ca or Sr [55]. The practical use of Na_x_CoO_2_ and Bi_2_A_2_Co_2_O_9_ is limited by the volatility of Na and Bi at high temperatures and the hygroscopicity of the compounds. Therefore, Ca_3_Co_4_O_9_ represents the most interesting layered cobaltite for thermoelectric applications, especially at high operating temperatures. Due to its structure, which consists of electron conducting layers separated by nonconducting layers that cause phonon scattering, the thermoelectric properties of Ca_3_Co_4_O_9_ are highly anisotropic. The in-plane direction is characterized by a high electrical conductivity within the CoO_2_ layers, resulting in a high power factor σ$\alpha^{2}$. However, in the out-of-plane direction, the phonon scattering is very high, resulting in an even lower thermal conductivity.

In general, the thermoelectric properties of bulk Ca_3_Co_4_O_9_ can be strongly influenced by the parameters of the synthesis and the sintering method, which have a strong influence on the resulting density, grain size, and orientation of the material [56]. Ca_3_Co_4_O_9_ can be synthesized via a solid-state mechanism or a sol-gel procedure. Krolicka et al. investigated the effect of these techniques on the structural and thermoelectric properties, and showed increased *zT* values in the sample prepared by the sol-gel route due to improved grain alignment [57]. The corresponding ceramics can be prepared by different sintering methods, such as spark plasma sintering (SPS) or pressureless sintering methods, all again leading to different grades of grain orientation and densification [56,58,59,60]. Bittner et al. showed that the porosity of a bulk Ca_3_Co_4_O_9_ ceramic strongly influences the resulting thermoelectric parameters [61]. A high porosity leads to a reduced electrical conductivity and simultaneously decreased thermal conductivity, thus improving the figure of merit *zT*. Therefore, the porosity as well as the synthesis method is one way to tune such oxide-based materials for an increased power factor (dense ceramic) or *zT* (high porosity).

As mentioned before, doping and nanostructuring are common ways to tune the thermoelectric parameters of thermoelectric materials. Table 1 shows the figure of merit *zT* and the power factor σ$\alpha^{2}$ of several doped Ca_3_Co_4_O_9_, Na_x_CoO_2_ and Bi_2_Ca_2_Co_2_O_9_ compounds. Here, Na_x_CoO_2_ exhibits the highest power factor, while Ca_3_Co_4_O_9_ is the most promising layered cobaltite when high efficiency and therefore high *zT* is desired. A nanocomposite containing all three layered cobaltites discussed here is also included, showing promising synergistic effects in its thermoelectric properties as well as its thermal stability [10].

Overall, the thermoelectric properties of layered cobaltites, and all oxide-based materials in general, strongly depend on many parameters, such as the synthesis route, morphology of the crystals, doping, nanostructuring, texturing, and densification into a bulk material, thus leading to a possible control of tuning oxide-based thermoelectric materials to a high power factor σ$\alpha^{2}$ or a high *zT* value for different application fields. However, more research on the enhancement of the power factor is required to increase the potential of these materials for high-temperature applications.

#### 2.1.2. *n*-Type Oxides

Around the same time as the first layered cobaltite compounds, other transition metal oxides were reported to have promising thermoelectric properties. Since then, the most studied oxide-based *n*-type materials have been the aforementioned ZnO, as well as SrTiO_3_ and CaMnO_3_. ZnO exhibits a hexagonal wurtzite structure with a large direct band gap of 3.44 eV [76], SrTiO_3_ has a cubic perovskite structure and is also characterized by a large band gap of 3.25 eV [77] and CaMnO_3_ crystallizes in an orthorhombic perovskite structure with an indirect band gap of 0.7 eV [78]. All these materials are characterized by a high Seebeck coefficient and a very low electrical conductivity due to a low carrier concentration without doping [9].

Based on the large band gap of undoped ZnO, increasing the carrier density by doping and defect engineering is used to ensure good thermoelectric properties. For ZnO, doping with Al has been widely studied and shows the highest *zT* values from 0.3 to 0.45 with moderate power factor values of approximately 5–8 μW cm^−1^ K^−2^ thus far [79,80,81]. Again, the synthesis parameters as well as the morphology of the crystals strongly influence the resulting thermoelectric parameters. Han et al. reported, that the *zT* value of ZnO with nanoparticle morphology is 1.5 times higher than that of a platelet-shaped morphology [82]. In addition to doping, defect engineering is another promising way to enhance the thermoelectric properties, as shown by Tian et al.; by increasing the Al solubility and therefore the carrier concentration and electrical conductivity, the thermal conductivity decreases due to introduced defects [83]. Undoped SrTiO_3_ also has a very large band gap, making it electrically insulating. However, by electron doping with group III elements (mostly lanthanides) on the Sr sites or group V elements (Nb, Ta) on the Ti sites, a strong increase in carrier density (up to approximately 10^20^ cm^−3^) and electrical conductivity (up to 50–1000 S cm^−1^) in single crystals can be observed [84]. In addition to doping and codoping with several lanthanides, the influences of Sr vacancies have been investigated and were reported to have a positive effect on thermoelectric properties [85,86]. Similarly to SrTiO_3_, CaMnO_3_ exhibits a high Seebeck coefficient but an electrically insulating character before doping. Here, rare-earth metals as dopants for the Ca sites as well as transition metals for the Ti sites have been investigated. CaMnO_3_ and SrTiO_3_ have perovskite structures with octahedral coordination of Mn and Ti, respectively. The symmetry of the MnO_6_ and TiO_6_ octahedrons also influences the resulting parameters. A distortion of the octahedron, e.g., that due to a Jahn–Teller distortion as a result of a partial reduction of Mn^4+^ to Mn^3+^ or due to doping with smaller or larger elements, influences the electrical and thermal conductivity of the material [9].

Additionally, *n*-type In_2_O_3_ was investigated as a promising oxide-based thermoelectric material [20,87]. Undoped In_2_O_3_ is a semiconductor with a band gap of 1.2 eV that can be strongly influenced via doping. The crystal structure can be described as a cubic bixbyite structure with two nonequivalent cation sites that can be substituted with different dopants [88]. The electron effective mass as well as the carrier concentration of this material strongly depend on the amount of doping within the structure. As a result, a very high carrier mobility can be achieved, making tuning the thermoelectric properties very promising [89]. Bittner et al. [20] presented Sn,Al-doped *n*-type In_2_O_3_, which reached a comparatively high power factor of 7.1 μW cm^−1^ K^−2^ at 1200 K. However, doped In_2_O_3_ suffers from its high thermal conductivity and thus shows a noteworthy *zT* only above 1000 K at this time. Nevertheless, doped In_2_O_3_ represents an interesting *n*-type thermoelectric material for high-temperature applications due to the high power factor resulting from high electrical conductivity.

Table 2 shows the figure of merit *zT* and the power factor σ$\alpha^{2}$ of several doped ZnO, SrTiO_3_, CaMnO_3_, and In_2_O_3_ bulk compounds. As described before, the thermoelectric properties also strongly depend on the synthesis method and the sintering parameters. Here, SrTiO_3_ compounds exhibit the highest power factor, while the other materials reach *zT* values of approximately 0.3–0.4, except for doped In_2_O_3_, which only reaches a *zT* value of 0.15.

### 2.2. BiCuSeO

**Figure 4:**
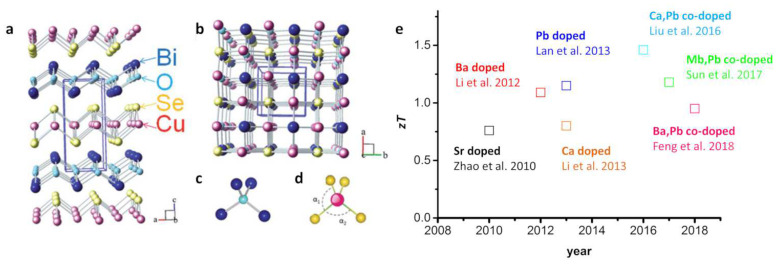
Crystal structure of BiCuSeO (**a**) along the b-axis and (**b**) along the c-axis; (**c**) Bi_4_O coordination tetrahedra, and (**d**) CuSe_4_ coordination tetrahedra. Reproduced from [50] with permission from The Royal Society of Chemistry; (**e**) *zT* values of various doped bulk BiCuSeO materials under an inert gas atmosphere [11,103,105,113,114,115,116].

Doped BiCuSeO is one of the newest and most promising thermoelectric materials. The first works presenting the thermoelectric properties of this compound were published from 2010 to 2012 [103,104,105]. BiCuSeO is one of several isostructural RMChO (R = Bi, Ce to Dy; M = Cu or Ag; Ch = S, Se or Te) compounds and exhibits a two-dimensional layered structure with Bi_2_O_2_ and Cu_2_Se_2_ layers, as shown in Figure 4a–d [50]. Due to this layered structure, BiCuSeO also shows anisotropic thermoelectric properties, which strongly depend on the synthesis method and the sintering parameters, analogous to the layered cobaltite compounds [50,106,107]. Due to the similar behavior between BiCuSeO and the oxide-based cobaltites, the former material is discussed here, although it is an oxyselenide and not an oxide material. BiCuSeO can be synthesized via hydrothermal methods, solid state reactions, sol-gel methods, or mechanical alloying. The bulk materials are again prepared via cold-pressing, hot-pressing, or SPS [50,51,108,109,110]. The electrical conductivity of undoped BiCuSeO is relatively low because of a low carrier concentration and carrier mobility, while the material exhibits an exceptionally high Seebeck coefficient of approximately 450 μV K^−1^ and a very low thermal conductivity of approximately 0.4 W m^−1^ K^−1^ [50,111]. Based on this, BiCuSeO can be described as a high-*zT* material but offers a relatively low power factor σ$\alpha^{2}$ comparable to those of the layered cobaltites. Although a very high *zT* > 1 can be reached. Additionally, BiCuSeO struggles with thermal stability in an air atmosphere showing surface oxidation at 573 K and complete decomposition at 773 K [112].

To improve the thermoelectric properties of BiCuSeO, adjusting the electrical conductivity by enhancing the carrier concentration via doping has been extensively studied. For this purpose, element doping with divalent cations at Bi sites to enhance the *p*-type electron conduction is very promising. Here, doping and codoping with various elements have been investigated. Figure 4e shows the reported peak *zT* of various doped BiCuSeO materials in recent years.

BiCuSeO is an intrinsic *p*-type semiconductor due to Bi and Cu vacancies. Recently, Pan et al. realized *n*-type BiCuSeO by iron incorporation [117] and Zhang et al. [118] presented the realization of *n*-type BiCuSeO by filling these vacancies with additional Bi and Cu and simultaneously introducing Br and I at the Se site for electron doping; the above resulted with Seebeck coefficients of up to −550 μV K^−1^.

### 2.3. Comparison of Oxides and Oxyselenides

**Figure 5:**
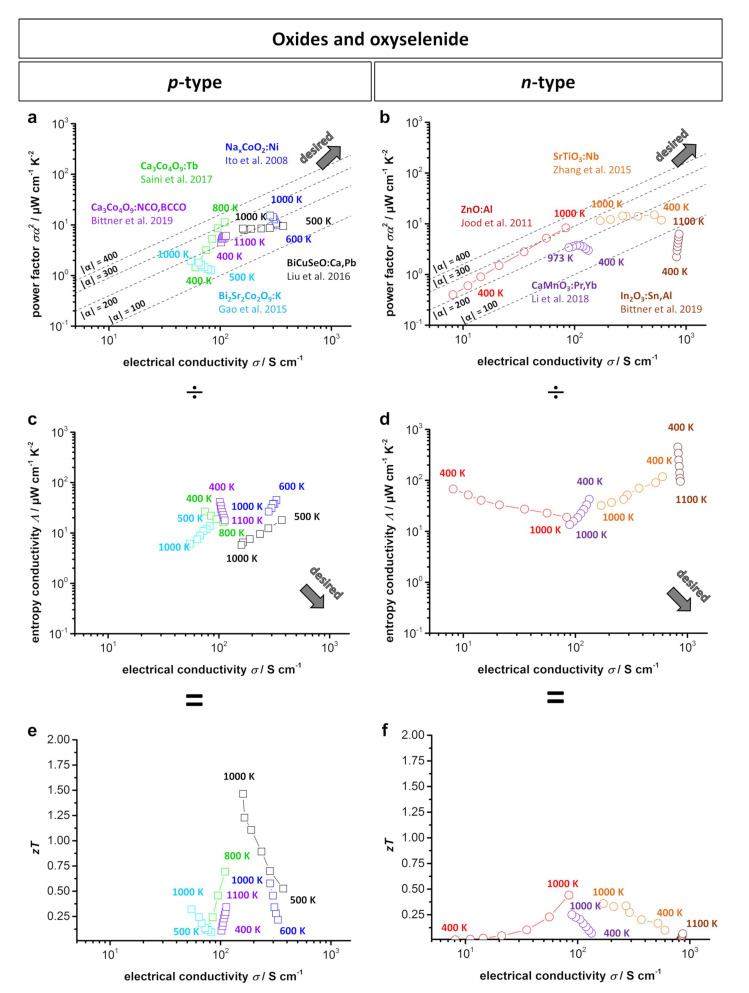
(**a**) *p*-Type; (**b**) *n*-type type-I Ioffe plots; (**c**) *p*-type; (**d**) *n*-type type-II Ioffe plots and (**e**) *p*-type and (**f**) *n*-type *zT*-electrical conductivity plots for several doped oxidic thermoelectric materials and BiCuSeO [10,11,39,69,74,80,96,102]. Dashed lines show the corresponding absolute values of the Seebeck coefficient α given in μV K^−1^. In the type-I Ioffe plot, a desired material would be located at a high power factor and simultaneously high electrical conductivity in the top right. In the type-II Ioffe plot, a desired material would be located at a low entropy conductivity and simultaneously high electrical conductivity in the bottom right. Note that the data in Ioffe plots of type-I and type-II can be divided by each other according to Equation (2) to give the dimensionless figure of merit *zT* as a function of the electrical conductivity.

Overall, the discussed oxide-based thermoelectric materials exhibit good *zT* values of approximately 0.2–0.8 with the oxyselenide compound BiCuSeO reaching *zT* values >1, while generally showing a medium power factor of 1–11 μW cm^−1^ K^−2^. Preparation parameters, doping, defect engineering, and nanostructuring can be utilized to tune the material behavior, thus enhancing the power factor or decreasing the thermal conductivity to reach higher *zT* values. Figure 5 shows the type-I and type-II Ioffe plots and *zT* plots for several of the doped *p*- and *n*-type oxide-based bulk materials and oxyselenides. Here, it can be easily seen that doped BiCuSeO and doped SrTiO_3_ exhibit the highest power factor in relation to electrical conductivity and are therefore the closest to the desired area. In comparison, Figure 5b displays the related *zT* values of the materials, where the BiCuSeO and Ca_3_Co_4_O_9_ reach the highest *zT* values. However, the BiCuSeO is not stable in an air atmosphere, as mentioned before. As oxide-based materials are of special interest for high-temperature applications, enhancement of the power factor and thus increasing the resulting power output must be further investigated.

## 3. Metals and Intermetallics

### 3.1. Zintl Phases

**Figure 6:**
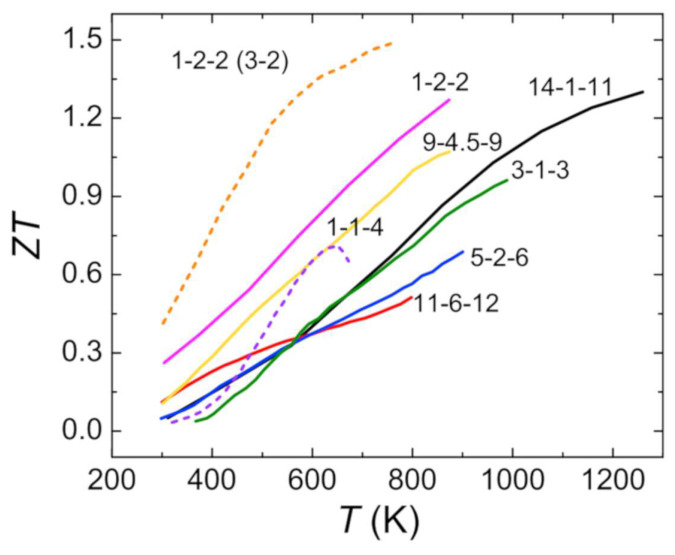
Temperature-dependent *zT*-values of Zintl phases with different stoichiometries. Solid lines represent *p*-type Zintl phases and dashed lines represent *n*-type Zintl phases. Different Zintl families are named after their respective stoichiometry (see also Table 3). Reused from [8] with permission from Elsevier.

Zintl phases are high-melting intermetallic compounds that are characterized by an ionic structure containing covalently bonded polyanions that build an ‘electron crystal’, while the cation layers act as a ‘phonon glass’. Although this form of intermetallics was discovered in the 1930s and has been highly investigated since, the first reports about their good thermoelectric properties were not published until 2005 [119]. The thermoelectric properties of many different Zintl families and structures have been investigated. The general composition of a Zintl phase can be described as A_a_BX_x_ with A = active, electropositive metal (mostly alkaline and earth alkaline metals); X = noble, electronegative metal from group 13, 14 and 15, and B = ternary transition metal (Zn, Cd, Mn). Based on this, different groups of Zintl phases can be named after their stoichiometry, e.g., 14-1-11 compounds such as Yb_14_MnSb_11_. In Figure 6 the *T*-dependent *zT* values of several *p*- and *n*-type Zintl groups can be seen. The investigated Zintl compounds exhibit varying *zT* values between 0.5 and 1.5. Due to their complex structure, Zintl phases are usually characterized by a very low glass-like thermal conductivity. The electronic structure strongly depends on the respective material, varying between extremely low carrier mobility and high carrier concentration in 0D 14-1-11 compounds, and very high carrier mobility and low carrier concentration in 2D 1-2-2 compounds [8]. In this section, several *p*-type (14-1-11, 5-2-6, 9-4.5-9, and 1-2-2) and the most recently investigated *n*-type 1-2-2 Zintl compounds will be discussed. Again, the reader is referred to other review articles for more details on the respective physical properties of Zintl phases [8,49,120].

#### 3.1.1. *p*-Type Zintl Phases

Currently, the best *p*-type Zintl family are the 14-1-11 compounds, reaching a *zT* > 1 at an operating temperature of up to 1200 K. These 14-1-11 compounds with the general formula A_14_MPn_11_ (A = alkaline-earth or rare earth element, M = Al, Mn, Zn, Ga, Nb, In or Cd and Pn = group 15 element) exhibit covalently bonded [MPn_4_]^9−^ tetrahedra and [Pn_3_]^7−^ linear components with Pn^3−^ and A^2+^ ions [121]. Due to their large unit cell and semiconductor nature with low electrical resistivity and low thermal conductivity, these compounds are very promising candidates for achieving good thermoelectric properties via doping. Within this family, Yb_14_MnSb_11_ showed the highest *zT* values of up to 1.3 [122]. Compared to its isostructural analog Yb_14_AlSb_11_, which was the first reported 14-1-11 Zintl compound, Yb_14_MnSb_11_ has the Al^3+^ replaced by Mn^2+^ resulting in *p*-type conduction. To optimize the extremely high carrier concentration of approximately 10^21^ cm^−3^, several different dopants at various sites have been investigated, e.g., La, Ca, Sc and Y at the Yb site [41,123,124] or Al and Mg at the Mn site [122]. Several doped 14-1-11 Zintl compounds are shown in Table 3. The resulting power factor is approximately 5–10 μW cm^−1^ K^−2^ and therefore, slightly higher than that of oxide-based materials. Yb_14_MnSb_11_ is usually prepared by a Sn-flux method or ball milling and densification via hot pressing or SPS sintering.

The 11-6-12 compounds are somewhat newly investigated thermoelectric materials. In 2014, the first work on the thermoelectric properties of *p*-type Eu_11_Cd_6_Sb_12_ was published [125]. The 11-6-12 compounds exhibiting a Sr_11_Cd_6_Sb_12_ structure type consist of [Cd_6_Sb_12_]^22−^ ribbons forming a 1D structure filled with Sr^2+^ cations [126] and are mostly prepared by the Sn-flux method. Due to the infinite 1D structure of polyanions, these compounds feature comparatively high thermal conductivity. For Zn-doped Sr_11_Cd_6_Sb_12_ a *zT* of 0.5 with a power factor of 5.6 μW cm^−1^ K^−2^ could be achieved [127]. The rather low power factor in 11-6-12 compounds is mainly due to the low carrier mobility of approximately 20–30 cm^2^ V^−1^ s^−1^ [127]. However, investigations of thermoelectric 11-6-12 have only started, and further optimization and tuning are still the focus of research. Similarly, the thermoelectric properties of 5-2-6 Zintl compounds were also recently reported. It can be described by the general formula A_5_M_2_Pn_6_ with A = alkaline earth or rare earth metal, M = trivalent metal and Pn = As, Sb or Bi. The 5-2-6 compounds are mainly produced via ball milling and hot pressing. Within this family, the two basic structure types are Ca_5_Ga_2_As_6_ and Ca_5_Al_2_Bi_6_, which both consist of infinite [M_2_Pn_6_]^10−^ chains and A^2+^ cations [128]. A peak *zT* of 0.7 with a power factor of 6.5 μW cm^−1^ K^−2^ can be reached in Zn-doped Ga_5_In_2_Sb_6_ [129]. Several other doped materials of the 11-6-12 and 5-2-6 families are shown in Table 3.

The 9-4+x-9 Zintl compounds with a general formula of A_9_M_4+x_Pn_9_ where A = Ca, Sr, Eu, or Yb, M = transition metal and Pn = Bi or Sb also consist of infinite ribbons of [M_4_Pn_9_]^19−^ components and exhibit partially filled interstitial sites filled with transition metal [130]. Therefore, the thermoelectric properties can be influenced by occupancy of the interstitial sites and by the exact stoichiometry [131]. To date, several doped and undoped compounds in this family have been investigated, including Yb_9_Mn_4+x_Sb_9_ [132], Eu_9_Cd_4+x_Sb_9_ [133], and Ca_9_Zn_4+x_Sb_9_ [131], reaching *zT* values of approximately 0.7 and a power factor of 5–7 μW cm^−1^ K^−2^ (compare Table 3). Here, again the Sn-flux technique as well as a combination of ball milling and hot pressing or SPS were utilized for preparation.

Next, to the 14-1-11 compounds, the family of 1-2-2 Zintl phases show very good thermoelectric properties, reaching a *zT* > 1. This group can be described as AB_2_X_2_ with A = Ca, Ba, Sr, Yb or Eu, B = Mn, Zn, Cd or Mg and X = As, Sb or Bi. The CaAl_2_Si_2_-type structure contains two-dimensional [B_2_X_2_]^2−^ sheets separated by A^2+^ cations [151]. Most *p*-type 1-2-2 Zintl phases are characterized by an extremely high *p*-type carrier concentration due to the vacancies on the A cation sites. Thus, substitution and doping at this site proves to be promising for tuning the thermoelectric properties. Subsequently, doped EuZn_2_Sb_2_ [143] and doped YbCd_2_Sb_2_ [144,145,146] reached *zT* values above *zT* = 1. Shuai et al. reported a *zT* value of 1.3 and a power factor of 13.5 μW cm^−1^ K^−2^ for Eu_0.5−x_Yb_0.5−x_Mg_2_Bi_2_ [149]. Additionally, binary *p*-type 1-2-2 compounds with A=B, e.g., Mg_3_Sb_2_, have been widely investigated [150,152]. However, due to their high resistivity, only a moderate *zT* at high temperatures can be reached. The thermoelectric properties of several ternary and binary 1-2-2 Zintl compounds are shown in Table 3. Similar to that of the other families, the 1-2-2 Zintl compounds are mostly prepared by melting or ball milling and densification via hot pressing or SPS.

#### 3.1.2. *n*-Type Zintl Phases

As described above, several *p*-type 1-2-2 compounds have been investigated resulting in *zT* values of up to 1.3. In this group, the stoichiometric Mg_3_Sb_2_ has taken a special role in research, due to its characteristically low carrier concentration, which opens a pathway to thermoelectric *n*-type Zintl phases [8]. Since the first report, in 2014, of *n*-type conduction in Mn-doped Mg_3_Sb_2_ [153], many different dopants have been investigated. Doping with Te as an electron donor and Bi to reduce the lattice thermal conductivity proved to be an effective way to realize high *zT* values in *n*-type Zintl phases. Most recently, Chen et al. [154] reached a *zT* value of 1.7 at a power factor of 20 μW cm^−1^ K^−2^ by combining Mn doping at the Mg site and Te and Bi doping at the Sb site. Table 4 gives an overview of several doped *n*-type Zintl phases based on Mg_3_Sb_2_. To date, *zT* values of approximately 1.5–1.7 and a power factor of up to 20 μW cm^−1^ K^−2^ have been reached.

#### 3.1.3. Comparison of Zintl phases

**Figure 7:**
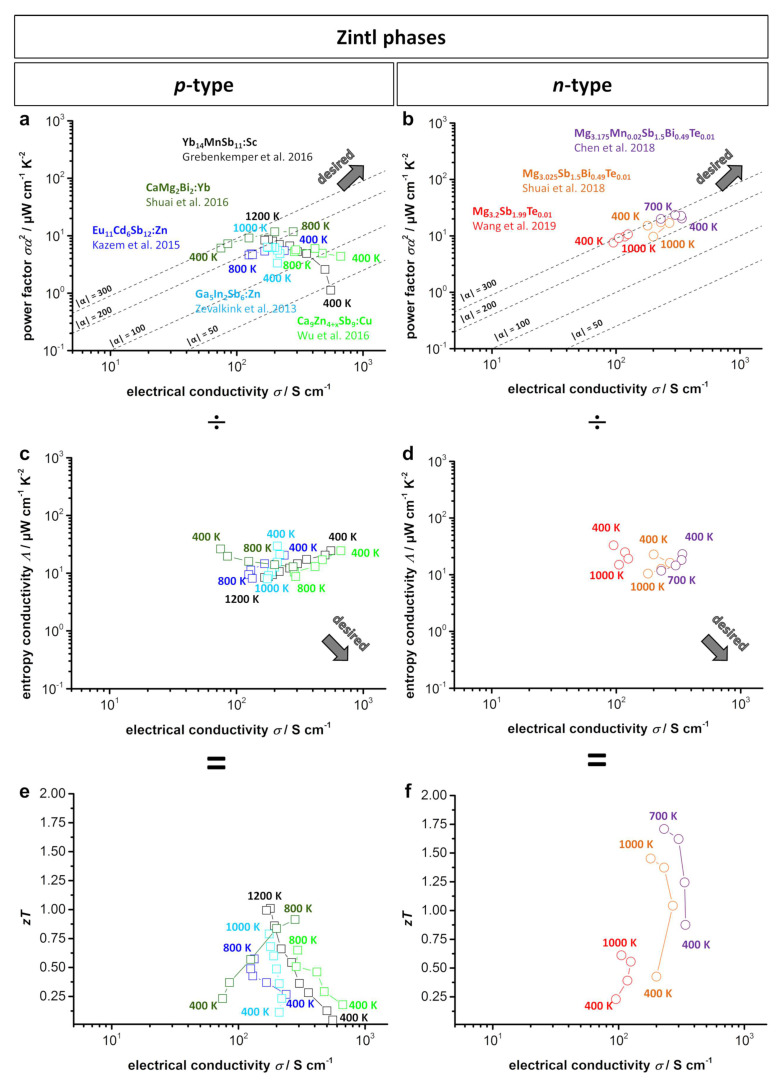
(**a**) *p*-Type; (**b**) *n*-type type-I Ioffe plots; (**c**) *p*-type; (**d**) *n*-type type-II Ioffe plots as well as (**e**) *p*-type and (**f**) *n*-type *zT*-electrical conductivity plots of several doped Zintl materials [41,127,129,131,148,154,155,157]. Dashed lines show the corresponding absolute values of the Seebeck coefficient α given in μV K^−1^.

Figure 7 shows the type-I and type-II Ioffe plots and *zT* plots from several of the doped *p*-type and *n*-type bulk Zintl materials. As described above, within the *p*-type Zintl phases, the 14-1-11 compounds show the best temperature stability up to 1200 K and reach the highest *zT* values. However, the Ioffe plots show that ternary 1-2-2 compounds and the 9-4+x-9 compounds feature a comparable or even higher power factor than 14-1-11 compounds at lower temperatures. Improvement of temperature stability could therefore lead to even higher power outputs at high operating temperatures. Within the *n*-type 1-2-2 Zintl phases, heavily doped Mg_3+δ_Sb_2_ exhibits the highest *zT* reported for Zintl phases thus far and a high power factor of approximately 20 μW cm^−1^ K^−2^. However, the operating temperature is limited to approximately 700 K for the heavily doped Mg_3+δ_Sb_2_ and up to 1000 K for previously reported compounds. Additionally, the Ioffe plots show a maximum power factor and electrical conductivity at approximately 700 K with decreasing values afterward. Enhancing the temperature stability and improving the thermoelectric properties at temperatures above 700 K may lead to a very promising *n*-type Zintl phase for application at high temperatures.

### 3.2. Heusler and Half-Heusler Compounds

**Figure 8:**
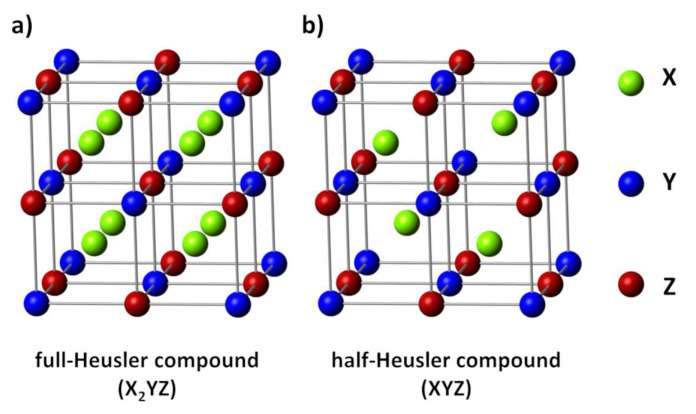
(**a**) Crystal structure of (**a**) full-Heusler- (X_2_YZ) and (**b**) half-Heusler-compounds (XYZ). The half-Heusler compounds exhibit an unoccupied sublattice of X resulting in promising thermoelectric characteristics.

Heusler compounds are intermetallics with the formula X_2_YZ and are characterized by their cubic structure with the space group *Fm*$\bar{3}$*m* [159]. The X and Y within this formula represent transition metals, while Z is a main group element. The half-Heusler compounds are derived from this and have the formula XYZ with the *F*$\bar{4}$3*m* space group [7,159]. The structures for both compounds are displayed in Figure 8. The full-Heusler structure (Figure 8a) can be described by four interpenetrating face-centered-cubic sublattices, where two of them are equally occupied by the X. In contrast, one of the equally occupied sublattices is vacant for the half-Heusler compounds (Figure 8b) [7]. The difference in the structures greatly affects the valence electrons and thereby the band structures of both compounds, typically leading to increased effective mass carrier concentrations and a high power factor for half-Heusler compounds, which is the reason for our focus on them within this review [159]. Half-Heusler materials are generally stable if 18 valence electrons are present, because only bonding states are occupied in this case [159]. Notably, this restricts the choice of elements for stable phases. The resulting phases usually show semiconducting behavior, e.g., with band gaps of 0.5 eV for XNiSn compounds or semimetallic behavior for (Zr,Hf)CoSb compounds [159,160,161]. This narrow electronic band structure results in a characteristically high power factor for half-Heusler compounds in comparison to other thermoelectric material classes [159]. Since the states near the Fermi level are mainly based on *d-d* orbital bonding, the density of states results in large Seebeck coefficients and high electrical conductivities [162]. A more detailed discussion of the physical properties of different half-Heusler compounds can be found in the respective review articles [35,37,49,159,163].

The first studies on half-Heusler compounds started in the early 1990s, but intensified with respect to thermoelectrics in the 2000s [164]. Recently, Poon presented an approach for dividing the advances of half-Heusler development for thermoelectrics into three different generations [163]. The first generation in the 2000s was characterized by alloying and doping as main modification factors providing *zT* values below 1, while in the second generation around 2010, more advanced synthesis techniques were introduced, namely, SPS for densification and nanostructuring, which led to *zT* values of approximately 1. In the current third generation, band engineering and structure ordering are becoming increasingly famous in addition to the previous techniques, which results in *zT* values of approximately 1.5 [163]. Advantages of half-Heusler compounds are their nontoxicity and stability to mechanical stress as well as high temperatures [7]. The optimum working temperature with regard to the thermoelectric performance is typically within 700–1000 K when in a vacuum or inert gas. The materials exhibit remarkable oxygen stability, but recent research has shown a sensitivity to oxygen at working temperatures for TiNiSn and ZrNiSn, resulting in the formation of oxides at the surface. Thus, the oxygen influence on the thermoelectric properties still needs to be investigated [165,166].

The elements of the half-Heusler compounds usually maintain very high melting points above 1773 K, which means high-temperature alloying, such as arc melting in a chamber with inert gas, is necessary for synthesis [162]. The usage of rather costly elements such as Hf further increases the production costs, which are major disadvantages of half-Heusler compounds despite their excellent thermoelectric properties.

#### 3.2.1. *p*-Type Half-Heusler Compounds

State-of-the-art *p*-type half-Heusler compounds are mainly based on FeNbSb, where Nb is substituted by Ti or Hf as shown in Table 5. By comparing the power factors of the compounds in Table 5, the high *zT* values were mainly reached by reducing the thermal conductivity with heavy dopants at relatively high doping amounts. The best properties were achieved for FeNb_0.88_Hf_0.12_Sb by Fu et al. [22] and reached a power factor of 51 μW cm^−1^ K^−2^ and a *zT* value of 1.45. However, a ZrCoBi-based compound recently reached *zT* values of up to 1.42, opening the way for research at a competing level. A similar half-Heusler system is based on XCoSb (X=Zr or Hf), which shows a decreased power factor at approximately 28 μW cm^−1^ K^−2^ but simultaneously a lower thermal conductivity compared to that of the FeNbSb-based compounds. The XCoSb compounds play a special role in thermoelectric research due to their possible *p*- and *n*-type doping, both leading to *zT* values of up to 1. [167,168] Therefore, these compounds can be found in both Table 5 and Table 6 depending on their respective dopants.

#### 3.2.2. *n*-Type Half-Heusler Compounds

Research on *n*-type half-Heusler compounds mainly focuses on MNiSn (M=Ti, Zr or Hf) compounds as parent materials. These compounds inherit quite remarkable *zT* values in the range of 0.4–0.55 as pure bulk materials, as shown in Table 6, but these values begin to rise to 1–1.5 when the materials are engineered properly toward improved thermoelectric performances. Reduction of grain size, alloying, and carrier doping are suitable tools to significantly improve the *zT* value [162]. Another parent material is XCoSb (X=Ti or Nb), which shows rather poor thermoelectric performance as a pure material due to high thermal conductivity, but drastically improves when doped [171,172]. The record *zT* value of 1.5 was reached with Ti_0.5_Zr_0.25_Hf_0.25_NiSn_0.998_Sb_0.002_ by Shutoh et al. [173]. Unfortunately, this value cannot be reproduced independently at this time; however, slightly lower values were achieved by similar chemical compositions [37]. As described for *p*-type half-Heusler materials, the XCoSb (X=Zr or Hf) compounds can also show *n*-type behavior when doped accordingly. Here, (Zr_0.4_Hf_0.6_)_0.88_Nb_0.12_CoSb shows a power factor of 27 μW cm^−1^ K^−2^ and a *zT* value of 0.99, as demonstrated by Liu et al. [168] in 2018.

#### 3.2.3. Comparison of Half-Heusler Compounds

**Figure 9:**
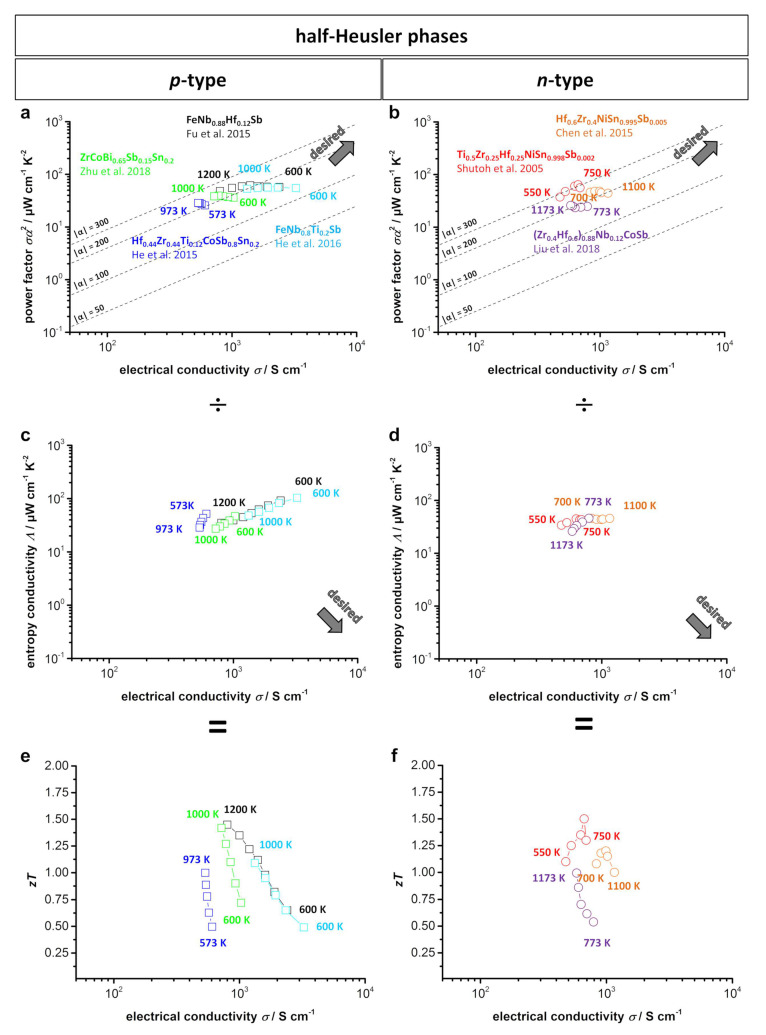
(**a**) *p*-Type; (**b**) *n*-type type-I Ioffe plots; (**c**) *p*-type; (**d**) *n*-type type-II Ioffe plots; (**e**) *p*-type; (**f**) *n*-type *zT*-electrical conductivity plots of several doped half-Heusler compounds [22,167,168,169,170,173,176]. Dashed lines show the corresponding absolute values of the Seebeck coefficient α given in μV K^−1^.

Figure 9 shows type-I and type-II Ioffe plots as well as *zT* plots from several of the doped *p*-type and *n*-type bulk half-Heusler materials. The comparatively high power factor that can be easily seen in the type-I Ioffe plots is attributed to the narrow electronic band structure and *d-d* orbital bonding, as already discussed. The half-Heusler compounds are therefore especially interesting for thermoelectric generators when a high power output is desired. A comparison of *p*- and *n*-type compounds shows similar results for the maximum values of the power factor of approximately 50 μW cm^−1^ K^−2^ and the *zT* up to 1.5, which results in no clear advantage of one specific type. However, the *p*-type compounds exhibit slightly higher thermal stability up to 1200 K for FeNbSb-based materials. The high power factor also means a rather high impact of thermal conductivity on the efficiency of a half-Heusler compound. Thus, further engineering is mainly targeted at reducing thermal conductivity.

### 3.3. SiGe Alloys

Eletrical supply of deep-space missions was one of the first application fields of thermoelectric power conversion. Here, mostly *n*- and *p*-type silicon–germanium alloys have been used [178]. This intermetallic material with a diamond crystal structure is characterized by a high thermal stability of up to 1200–1300 K when tested in vacuum. While Si exhibits a very high thermal conductivity of 148 W m^−1^ K^−1^, the introduction of Ge atoms in the Si matrix strongly enhances the phonon scattering, resulting in a thermal conductivity of approximately 2–5 W m^−1^ K^−1^ and a *zT* > 1 in nanostructured SiGe alloys [120]. Achieving *p*- and *n*-type conduction is realized via doping with B [40] or Ga [179] (*p*-type) and P [180] or Sb [181] (*n*-type). The thermoelectric properties can be strongly influenced by the exact stoichiometry of the SiGe alloy. Materials with an ideal ratio of Si_80_Ge_20_ have been found and widely studied. In SiGe alloys, the large difference in the mean free path between electron (approximately 5 nm) and phonon (approximately 200–300 nm) contributions leads to a strong influence of nanostructuring in a range of 10–100 nm, which reduces the thermal conductivity without significantly reducing the electrical conductivity [182]. Therefore, nanostructuring [183,184,185] and the use of nanoinclusions [184,186,187,188,189] are common strategies to further improve the thermoelectric properties of SiGe alloys. For preparation of SiGe alloys, solid-state ball milling [180,183,186,187,190,191,192,193,194] or melt spinning (MS) [182,189] in combination with subsequent SPS are commonly used. Bathula et al. [192] reported a peak *zT* of 1.72 with a power factor of 28.7 μW cm^−1^ K^−2^ for *n*-doped Si_80_Ge_20_ with SiC nanoinclusions. In 2016, Ahmad et al. [191] presented a strong increase in *p*-type Si_80_Ge_20_ performance up to a *zT* of 1.81 and a power factor of 39.05 μW cm^−1^ K^−2^ via Y_2_O_3_ nanoinclusions. Table 7 shows the *zT* value and power factor of several *n*- and *p*-type doped Si_80_Ge_20_ materials.

**Figure 10:**
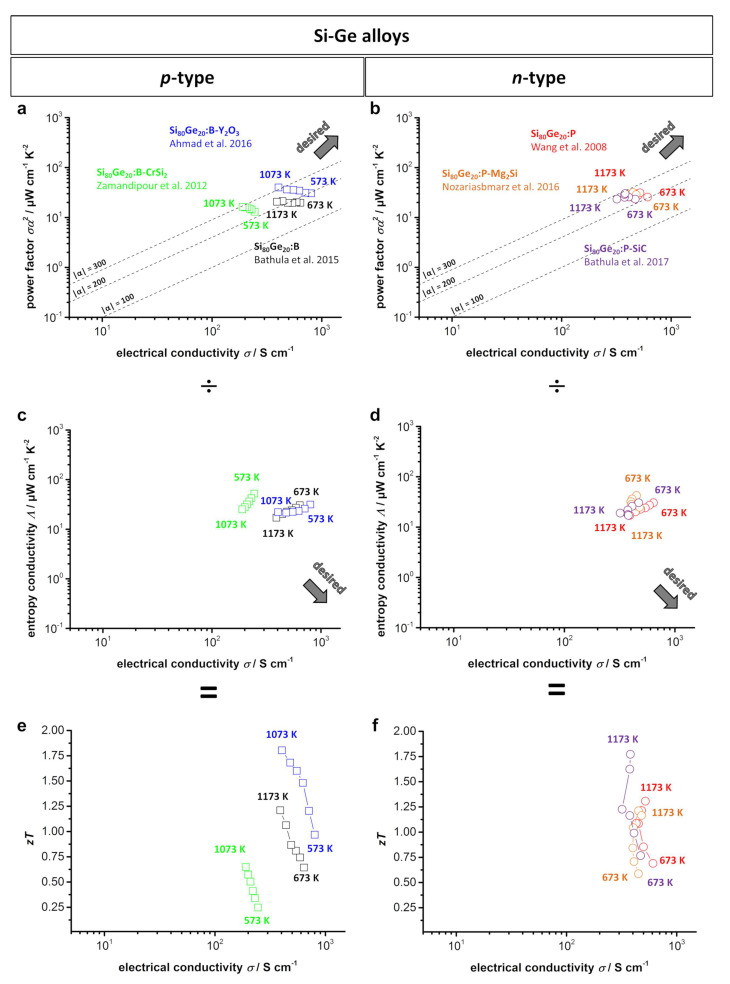
(**a**) *p*-Type; (**b**) *n*-type type-I Ioffe plots; (**c**) *p*-type; (**d**) *n*-type type-II Ioffe plots; (**e**) *p*-type; (**f**) *n*-type *zT*-electrical conductivity plots of several doped Si-Ge alloys [40,185,186,191,192,193]. Dashed lines show the corresponding absolute values of the Seebeck coefficient α given in μV K^−1^.

Figure 10 shows the type-I and type-II Ioffe plots and *zT* plots from several doped Si_80_Ge_20_ alloys. In general, Si-Ge alloys are characterized by a relatively high power factor of approximately 15–40 μW cm^−1^ K^−2^ due to their high electrical conductivity, which is why they are located at the top right of the type-I Ioffe plot. The drawback of a simultaneously high thermal conductivity can be mitigated via nanostructuring and nanoinclusions without significantly reducing the electrical conductivity, resulting in *zT* values of up to 1.7 for *n*-type and 1.8 for *p*-type materials. Combined with a high thermal stability of up to 1200–1300 K in vacuum, Si-Ge alloys are perfect candidates as thermoelectric materials for deep-space missions. Further adjustment of the thermal conductivity of such alloys is especially interesting if a high conversion efficiency is desired.

## 4. Comparison of High-Temperature Thermoelectric Materials

**Figure 11:**
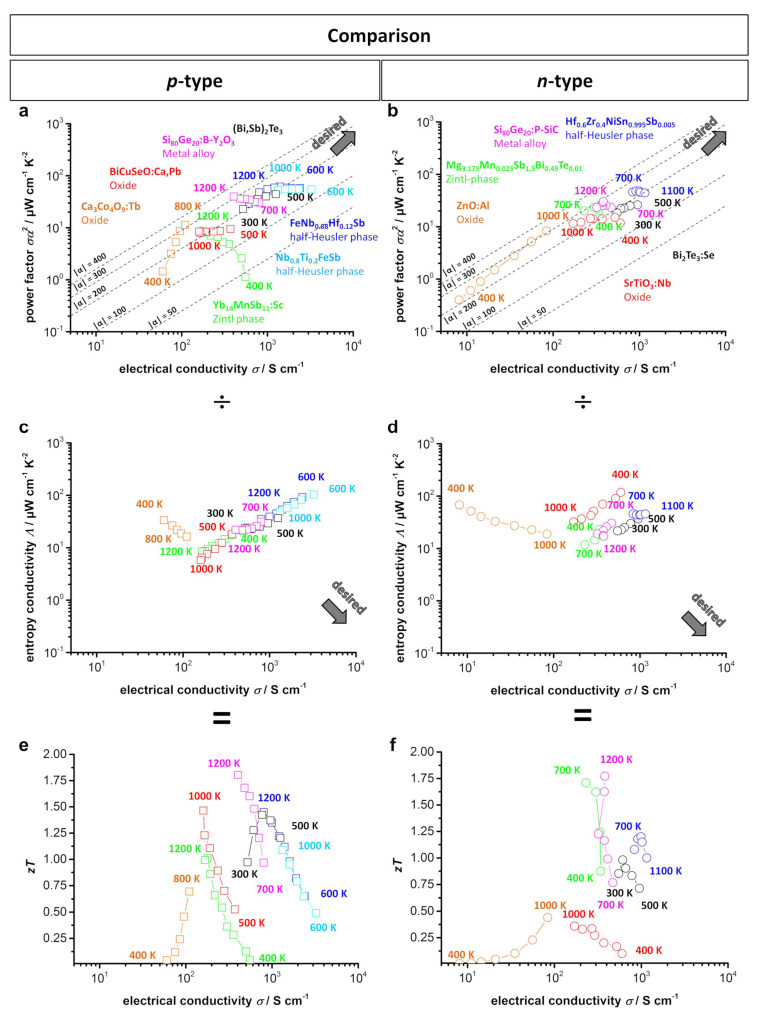
(**a**) *p*-Type; (**b**) *n*-type type-I Ioffe plots; (**c**) *p*-type; (**d**) *n*-type type-II Ioffe plots; (**e**) *p*-type; (**f**) *n*-type *zT*-electrical conductivity plots of several doped oxide materials [11,39,80,96], Zintl phases [41,154], half-Heusler compounds [169,173,176], and Si-Ge alloys [191,192] as compared to doped Bi_2_Te_3_ [21,195]. Dashed lines show the corresponding absolute values of the Seebeck coefficient α given in μV K^−1^.

Figure 11 shows the type-I and type-II Ioffe plots and *zT* plots from several of the doped *p*- and *n*-type materials for possible high temperature applications and their comparison to those of the commercially used Bi_2_Te_3_. Here, the half-Heusler compounds exhibit the highest power factor values with a simultaneously high electrical conductivity as a result of their electronic band structure, described above. Therefore, the half-Heusler compounds are the closest to the desired area for both *p*- and *n*-type materials. The conventional Bi_2_Te_3_ and the SiGe alloys also show a comparably high power factor at a slightly lower electrical conductivity. Of the compared thermoelectric materials, the oxide-based materials have the lowest power factor and electrical conductivity. This trend corresponds to the type-II Ioffe plots, where the half-Heusler compounds and Bi_2_Te_3_ exhibit the highest entropy conductivity, while the oxide materials show a significantly lower entropy conductivity, especially at high temperatures. Furthermore, the Zintl phases are also characterized by a low thermal conductivity, which culminates in the *n*-type Zintl phase of Mg_3.175_Mn_0.025_Sb_1.5_Bi_0.49_Te_0.01_ having the lowest entropy conductivity of all compared *n*-type materials. As a result, the half-Heusler compounds as well as BiCuSeO show the highest *zT* values within the *p*-type materials, and the Zintl compounds have the highest *zT* value within the *n*-type materials. As described before, the power factor corresponds to the maximum power output of the material, making the half-Heusler compounds the most interesting bulk materials for high-temperature application from this point of view. Yet, the Zintl compounds and oxyselenides provide a high efficiency in power conversion due to their significantly lower entropy conductivity. The oxide-based thermoelectric materials show comparatively low thermoelectric properties, but are characterized by high chemical and thermal stability, especially in air. As mentioned within the introduction, extension to a functional device always comes alongside additional tasks. In particular, contact resistivity is crucial to reach full potential, when applying highly electrical conducting materials such as half-Heusler compounds in a device.

**Figure 12:**
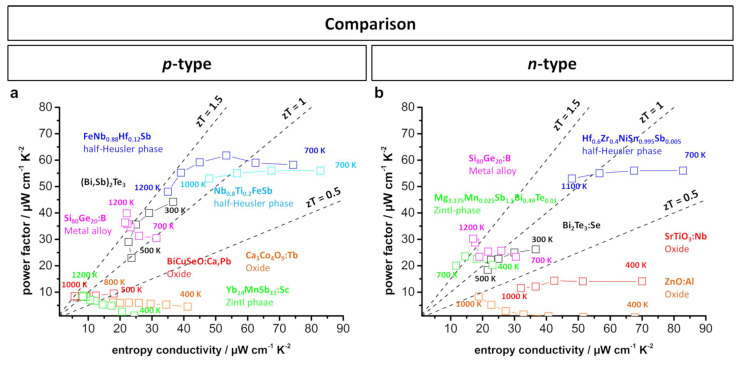
(**a**) *p*-Type; (**b**) *n*-type power factor vs. entropy conductivity plots of several doped oxide materials [11,39,80,96], Zintl phases [41,154], half-Heusler compounds [169,176,196] and Si-Ge alloys [191,192] as compared to doped Bi_2_Te_3_ [21,195]. Dashed lines show the corresponding *zT* values.

A similar comparison of the material properties can be performed by displaying the power factor as a function of the entropy conductivity, shown in Figure 12. Note that the dashed lines within the plot represent the dimensionless *zT* value. It can be easily observed that within the *p*-type thermoelectric materials, several compounds from different classes, such as BiCuSeO, SiGe alloys, and half-Heusler alloys, all reach a *zT* value of up to 1.5, whereas the power factor shows a strong deviation between 10 and 65 μW cm^−1^ K^−2^. Within the compared *n*-type materials, the same behavior can be observed, although the materials show overall slightly lower *zT* values. Therefore, this power factor vs. entropy conductivity plot presents the respective advantages of each kind of thermoelectric material discussed in terms of a high power factor or high *zT* value.

In addition to the thermoelectric materials discussed above, there are a few material classes with noteworthy thermal stability of up to 800–900 K, which should also be mentioned here. Cage compounds such as clathrates [197] and skutterudites [198] are both characterized by good electronic transport properties while reducing thermal conductivity by filling the cages with guest atoms [49]. In this way, high *zT* values of up to 1.3 and 1.7 at 800 K can be reached in multiple filled clathrates [199] and skutterudites [200,201], respectively. However, despite the similarity of these cage compounds, the respective power factor shows a strong variation with approximately 10 μW cm^−1^ K^−2^ for clathrates [199] and approximately 50 μW cm^−1^ K^−2^ for skutterudites [201] at 800 K. This also results in a possible tuning for either a high power output or a high conversion efficiency. Last, solid solutions of Mg_2_Si intermetallic silicides are also a focus of interest as mid-temperature thermoelectric materials of up to 800 K [49]. Solid solutions of Mg_2_Si_1−x_Ge_x_ and Mg_2_Si_1−x_Sn_x_, with an optimized carrier concentration via doping, reached a power factor of approximately 30 μW cm^−1^ K^−2^ and a *zT* value > 1 at 800 K [202,203]. For respective applications at mid-temperatures, a similar comparison to this work could be performed for evaluation and comparison of these materials and their respective parameters.

## 5. Conclusions

Different kinds of bulk thermoelectric materials have been compared with respect to their high-temperature performance and stability. Within the respective thermoelectric material classes, much research has been conducted within recent decades, however, very few works have compared these classes. Here, the concept of using Ioffe plots to compare and evaluate the power factor and the *zT* value as two different parameters that can be useful for optimization was successfully presented. Hereby, the strengths and weaknesses of each material class were revealed, which could be useful for prospective research and associated applications. Out of all of the compared materials, the class of half-Heusler compounds exhibited the highest power factor and electrical conductivity, which is applicable for reaching a high power output at high operating temperatures. Si-Ge alloys reached the highest *zT* values but had a significantly lower power factor than the half-Heusler compounds. Other materials, such as oxide-based materials, oxyselenides, and Zintl compounds, also reached reasonable *zT* values, which made these promising materials for reaching high conversion efficiencies.

## Figures and Tables

**Table 1:** Dimensionless figure of merit *zT* and power factor σ$\alpha^{2}$ of *p*-type Ca_3_Co_4_O_9_, Na_x_CoO_2_ and Bi_2_Ca_2_Co_2_O_9_ with different dopants.

Material	Dopant	*T*/K	*zT*	σ$\alpha^{2}$/μW cm^−1^ K^−2^
Ca_3_Co_4_O_9_	Cr [62]	1000 K	0.16	3.5
	Sm [63]	1000 K	0.15	2.4
	Tb [39]	1000 K	0.73	11.5
	K [64]	1000 K	0.22	2.85
	Cd [65]	1000 K	0.35	5.25
	Sr [66]	1000 K	0.22	3.95
	Na,W codopants [67]	1000 K	0.21	2.7
	La,Fe codopants [68]	1000 K	0.32	4.15
	NCO,BCCO nanocomposite [10]	1100 K	0.34	6.08
Na_x_CoO_2_	Ag,Au [69]	1000 K	0.4–0.5	13–15
	Ni [70]	1073 K	-	10.8
	Cu [71]	1000 K	-	15.5
	K, Sr, Y, Nd, Sm, Yb [72]	1000 K	0.36–0.5	6.8–7.3
Bi_2_Ca_2_Co_2_O_9_	Na [73]	900 K	-	2.1
	K [74]	1000 K	0.305	1.92
	Pb,La [75]	1000 K	-	1.6–2.2

**Table 2:** Figure of merit *zT* and power factor σ$\alpha^{2}$ of *n*-type ZnO, SrTiO_3_, CaMnO_3_, and In_2_O_3_ with different dopants.

Material	Dopant	*T*/K	*zT*	σ$\alpha^{2}$/μW cm^−1^ K^−2^
ZnO	Al [79,80,81]	1073	0.3–0.45	5–8
	Ni [90]	1073	0.09	5.8
	Al,Ni codopants [91]	773	0.06	5.6
	Ga [92]	973	0.25	12
SrTiO_3_	La,Nb,Sm,Gd,Dy [93]	1073	0.2–0.28	5.5–9
	La [94]	973	0.365	11.6
	Gd [95]	1023	0.37	10.9
	Nb [96]	1023	0.39	11.3
	Nb,Nd codopants [97]	1073	0.315	8.8
CaMnO_3_	Nb [98]	1073	0.325	1.9
	Dy,Ho,Er,Yb [99]	1000	0.15–0.2	2–3.5
	W [100]	1073	0.16	3.2
	Y,Dy codopants [101]	800	0.18	3.1
	Pr,Yb codopants [102]	973	0.24	3.3
In_2_O_3_	Sn,Al [20]	1200	0.08	7.1
	Ge,Mn,Zn [20]	1200	0.15	3.6

**Table 3:** Dimensionless figure of merit *zT* and power factor σ$\alpha^{2}$ of several *p*-type Zintl phases with different dopants.

Composition	Materials	Dopant	*T*/K	*zT*	σ$\alpha^{2}$/μW cm^−1^ K^−2^
14-1-11	Yb_14_MnSb_11_ [121]	-	1200 K	1.02	6.1
		Al [122]	1200 K	1.28	8.82
		Sc [41]	1200 K	1.02	8.38
		Y [41]	1200 K	1.01	6.85
	Yb_14_MgSb_11_ [134]	-	1200 K	1.03	6.5
	Sr_14_MgBi_11_ [135]	-	1200 K	0.71	9.5
11-6-12	Eu_11_Cd_6_Sb_12_	Zn [127]	800 K	0.51	5.55
		As [125]	800 K	0.185	1.69
5-2-6	Ca_5_Al_2_Sb_6_	Na [136]	1050 K	0.605	4.44
		Zn [137]	800 K	0.4	3.75
		Mn [138]	850 K	0.42	4.12
	Ga_5_In_2_Sb_6_	Zn [129]	950 K	0.72	6.56
	Eu_5_In_2_Sb_6_	Zn [139]	800 K	0.28	4.08
		Cd [140]	850 K	0.46	5.2
	Sr_5_In_2_Sb_6_	Zn [141]	800 K	0.36	4.13
9-4+x-9	Yb_9_Mn_4.2_Sb_9_ [132]	-	1000 K	0.74	4.53
	Ca_9_Zn_4+x_Sb_9_	Cu [131]	850 K	0.71	6.72
1-2-2	CaZn_2_Sb_2_	Na,Mg [142]	800 K	0.85	9.24
	EuZn_2_Sb_2_	Cd [143]	650 K	1.05	22.5
	YbCd_2_Sb_2_	Mn [144]	650 K	1.13	10
		Zn [145]	700 K	1.22	19.2
		Mg [146]	650 K	1.06	16.8
	CaMg_2_Bi_2_	Na [147]	850 K	0.88	12.3
		Yb [148]	850 K	0.96	12.2
	Eu_0.5−x_Yb_0.5−x_Mg_2_Bi_2_	Ca [149]	850 K	1.26	13.5
	Mg_3_Sb_2_	Na [150]	750 K	0.58	4.2

**Table 4:** Dimensionless figure of merit *zT* and power factor σ$\alpha^{2}$ of several *n*-type Zintl phases with different dopants.

Basis	Material	*T*/K	*zT*	σ$\alpha^{2}$/μW cm^−1^ K^−2^
Mg_3_Sb_2_	Mg_3+δ_Sb_1.99_Te_0.01_ [155]	700 K	0.61	9.16
	Mg_3+δ_Sb_1.48_Bi_0.48_Te_0.04_ [156]	700 K	1.59	12.56
	Mg_3+δ_Sb_1.48_Bi_0.49_Te_0.01_ [157]	700 K	1.45	15.14
	Mg_3+δ_Nb_0.15_Sb_1.5_Bi_0.49_Te_0.01_ [158]	700 K	1.52	18.5
	Mg_3+δ_Mn_0.025_Sb_1.5_Bi_0.49_Te_0.01_ [154]	700 K	1.71	20.02

**Table 5:** Dimensionless figure of merit *zT* and power factor σ$\alpha^{2}$ of several *p*-type half-Heusler phases.

Material	*T*/K	*zT*	σ$\alpha^{2}$/μW cm^−1^ K^−2^
FeNb_0.88_Hf_0.12_Sb [22]	1200 K	1.45	51
FeNb_0.86_Zr_0.14_Sb [22]	1050 K	0.80	46
FeNb_0.95_Ti_0.05_Sb [169]	973 K	0.70	50
FeNb_0.8_Ti_0.2_Sb [169]	973 K	1.10	53
ZrCoBi_0.65_Sb_0.15_Sn_0.20_ [170]	973 K	1.42	38
Hf_0.44_Zr_0.44_Ti_0.12_CoSb_0.8_Sn_0.2_ [167]	973 K	1	28

**Table 6:** Dimensionless figure of merit *zT* and power factor σ$\alpha^{2}$ of several *n*-type half-Heusler phases.

Material	*T*/K	*zT*	σ$\alpha^{2}$/μW cm^−1^ K^−2^
TiNiSn [174]	775 K	0.4	24
ZrNiSn [175]	1000 K	0.55	33
HfNiSn [175]	1000 K	0.48	35
Ti_0.5_Zr_0.25_Hf_0.25_NiSn_0.998_Sb_0.002_ [173]	700 K	1.50	62
Hf_0.6_Zr_0.4_Hf_0.25_NiSn_0.995_Sb_0.005_ [176]	900 K	1.20	47
NbCoSb_0.8_Sn_0.2_ [172]	973 K	0.56	21
TiFe_0.15_Co_0.85_Sb [177]	850 K	0.45	22
(Zr_0.4_Hf_0.6_)_0.88_Nb_0.12_CoSb [168]	1173 K	0.99	27

**Table 7:** Dimensionless figure of merit *zT* and power factor σ$\alpha^{2}$ of several doped *p*- and *n*-type SiGe alloys.

Composition	Dopant	Inclusion	*T*/K	*zT*	σ$\alpha^{2}$/μW cm^−1^ K^−2^
*n*-type Si_80_Ge_20_	P [190]	-	1073	1.78	30.3
	P [185]	-	1173	1.3	30.61
	Sb [181]	-	1073	0.61	18.5
	P	SiC [192]	1173	1.72	28.74
	P	Mg_2_Si [193]	1173	1.27	29.84
	P	FeSi_2_ [194]	1173	1.18	27.8
	P	WSi_2_ [187]	1173	1.16	35.27
*p*-type Si_80_Ge_20_	B [40]	-	1173	1.22	20.5
	B [180]	-	1073	0.96	22
	Ga [179]	-	1073	0.52	15.5
	B	Y_2_O_3_ [191]	1073	1.81	39.05
	B	CrSi_2_ [186]	1073	0.65	21.25
	B	YSi_2_ [189]	1073	0.53	16.57
	B	WSi_2_ [187]	1173	0.66	17.63

## References

[1] He J., Tritt T.M. (2017). Advances in Thermoelectric Materials Research: Looking Back and Moving Forward. Science.

[2] (2018). Energy Flow Chart U.S. 2017.

[3] Kishore R.A., Marin A., Wu C., Kumar A., Priya S. (2019). Energy Harvesting—Materials, Physics, and System Design with Practical Examples.

[4] Goldsmid H.J. (2010). Introduction to Thermoelectricity.

[5] Ioffe A.F. (1957). Semiconductor Thermoelements, and Thermoelectric Cooling.

[6] Snyder G.J., Toberer E. (2008). Complex Thermoelectric Materials. Nat. Mater..

[7] Chen S., Ren Z. (2013). Recent Progress of half-Heusler for Moderate Temperature Thermoelectric Applications. Mater. Today.

[8] Shuai J., Mao J., Song S., Zhang Q., Chen G., Ren Z. (2017). Recent Progress and Future Challenges on Thermoelectric Zintl Materials. Mater. Today Phys..

[9] Yin Y., Tudu B., Tiwari A. (2017). Recent Advances in Oxide Thermoelectric Materials and Modules. Vacuum.

[10] Bittner M., Kanas N., Hinterding R., Steinbach F., Groeneveld D., Wemhoff P., Wiik K., Einarsrud M.A., Feldhoff A. (2019). Triple-phase Ceramic 2D Nanocomposite with Enhanced Thermoelectric Properties. J. Eur. Ceram. Soc..

[11] Liu Y., Zhao L.D., Zhu Y., Liu Y., Li F., Yu M., Liu D.B., Xu W., Lin Y.H., Nan C.-W. (2016). Synergistically Optimizing Electrical and Thermal Transport Properties of BiCuSeO via a Dual-Doping Approach. Adv. Energy Mater..

[12] Zhao L.D., Lo S.H., Zhang Y., Sun H., Tan G., Uher C., Wolverton C., Dravid V.P., Kanatzidis M.G. (2014). Ultralow Thermal Conductivity and High Thermoelectric Figure of Merit in SnSe Crystals. Nature.

[13] Toshima N. (2017). Recent Progress of Organic and Hybrid Thermoelectric Materials. Synth. Met..

[14] Wolf M., Menekse K., Mundstock A., Hinterding R., Nietschke F., Oeckler O., Feldhoff A. (2019). Low Thermal Conductivity in Thermoelectric Oxide-Based Multiphase Composites. J. Electron. Mater..

[15] Feldhoff A. (2015). Thermoelectric Material Tensor Derived from the Onsager–de Groot–Callen Model. Energy Harvest. Syst..

[16] Fuchs H.U. (2014). A Direct Entropic Approach to Uniform and Spatially Continuous Dynamical Models of Thermoelectric Devices. Energy Harvest. Syst..

[17] Job G., Rüffler R. (2014). Physical Chemistry from a Different Angle.

[18] Goupil C., Seifert W., Zabrocki K., Müller E., Snyder G.J. (2011). Thermodynamics of Thermoelectric Phenomena and Applications. Entropy.

[19] Fuchs H. (2010). The Dynamics of Heat—A Unified Approach to Thermodynamics and Heat Transfer.

[20] Bittner M., Kanas N., Hinterding R., Steinbach F., Räthel J., Schrade M., Wiik K., Einarsrud M.A., Feldhoff A. (2019). A Comprehensive Study on Improved Power Materials for High-Temperature Thermoelectric Generators. J. Power Sources.

[21] Poudel B., Hao Q., Ma Y., Lan Y., Minnich A., Yu B., Yan X., Wang D., Muto A., Vashaee D. (2008). High-Thermoelectric Performance of Nanostructured Bismuth Antimony Telluride Bulk Allys. Science.

[22] Fu C., Bai S., Liu Y., Tang Y., Chen L., Zhao X., Zhu T. (2015). Realizing High Figure of Merit in Heavy-Band p-Type half-Heusler Thermoelectric Materials. Nat. Commun..

[23] Narducci D. (2011). Do we Really need High Thermoelectric Figures of Merit? A Critical Appraisal to the Power Conversion Efficiency of Thermoelectric Materials. Appl. Phys. Lett..

[24] Pei Y., Shi X., Lalonde A., Wang H., Chen L., Snyder G.J. (2011). Convergence of Electronic Bands for High Performance Bulk Thermoelectrics. Nature.

[25] Pei Y., Wang H., Snyder G.J. (2012). Band Engineering of Thermoelectric Materials. Adv. Mater..

[26] Hu L., Zhu T., Liu X., Zhao X. (2014). Point Defect Engineering of High-Performance Bismuth-Telluride-Based Thermoelectric Materials. Adv. Funct. Mater..

[27] Kanatzidis M.G. (2010). Nanostructured Thermoelectrics: The New Paradigm?. Chem. Mater..

[28] Cook B.A., Kramer M.J., Harringa J.L., Han M.K., Chung D.Y., Kanatzidis M.G. (2009). Analysis of Nanostructuring in High Figure-of-Merit Ag_1−x_Pb_m_SbTe_2+m_ Thermoelectric Materials. Adv. Funct. Mater..

[29] Li J., Tan Q., Li J.F., Liu D.W., Li F., Li Z.Y., Zou M., Wang K. (2013). BiSbTe-based Nanocomposites with High zT: The Effect of SiC Nanodispersion on Thermoelectric Properties. Adv. Funct. Mater..

[30] Lan Y., Minnich A.J., Chen G., Ren Z. (2010). Enhancement of Thermoelectric Figure-of-Merit by a Bulk Nanostructuring Approach. Adv. Funct. Mater..

[31] Miyazaki K., Kuriyama K., Yabuki T. Printable Thermoelectric Device. Proceedings of the PowerMEMS 2018 Conference.

[32] Biswas K., He J., Blum I.D., Wu C.I., Hogan T.P., Seidman D.N., Dravid V.P., Kanatzidis M.G. (2012). High-Performance Bulk Thermoelectrics with All-Scale Hierarchical Architectures. Nature.

[33] Zheng Y., Zhang Q., Su X., Xie H., Shu S., Chen T., Tan G., Yan Y., Tang X., Uher C. (2015). Mechanically Robust BiSbTe Alloys with Superior Thermoelectric Performance: A Case Study of Stable Hierarchical Nanostructured Thermoelectric Materials. Adv. Energy Mater..

[34] Tritt T.M. (2004). Thermal Conductivity—Theory, Properties and Applications.

[35] Tan G., Zhao L.D., Kanatzidis M.G. (2016). Rationally Designing High-Performance Bulk Thermoelectric Materials. Chem. Rev..

[36] Fergus J.W. (2012). Oxide Materials for High Temperature Thermoelectric Energy Conversion. J. Eur. Ceram. Soc..

[37] Poon S. (2018). Recent Advances in Thermoelectric Performance of half-Heusler Compounds. Metals.

[38] Cowen L.M., Atoyo J., Carnie M.J., Baran D., Schroeder B.C. (2017). Review—Organic Materials for Thermoelectric Energy Generation. ECS J. Solid State Sci. Technol..

[39] Saini S., Yaddanapudi H.S., Tian K., Yin Y., Magginetti D., Tiwari A. (2017). Terbium Ion Doping in Ca_3_Co_4_O_9_: A Step Towards High-Performance Thermoelectric Materials. Sci. Rep..

[40] Bathula S., Jayasimhadri M., Gahtori B., Singh N.K., Tyagi K., Srivastava A.K., Dhar A. (2015). The Role of Nanoscale Defect Features in Enhancing the Thermoelectric Performance of p-Type Nanostructured SiGe Alloys. Nanoscale.

[41] Grebenkemper J.H., Klemenz S., Albert B., Bux S.K., Kauzlarich S.M. (2016). Effects of Sc and Y Substitution on the Structure and Thermoelectric Properties of Yb_14_MnSb_11_. J. Solid State Chem..

[42] Bell L.E. (2008). Cooling, Heating, Generating Power, and Recovering Waste Heat with Thermoelectric Systems. Science.

[43] Kim H.S., Liu W., Ren Z. (2017). The Bridge Between the Materials and Devices of Thermoelectric Power Generators. Energy Environ. Sci..

[44] Snyder G.J., Snyder A.H. (2017). Figure of Merit zT of a Thermoelectric Device Defined from Materials Properties. Energy Environ. Sci..

[45] Tan G., Ohta M., Kanatzidis M.G. (2019). Thermoelectric Power Generation: From New Materials to Devices. Philos. Trans. R. Soc. A Math. Phys. Eng. Sci..

[46] He J., Liu Y., Funahashi R. (2011). Oxide Thermoelectrics: The Challenges, Progress, and Outlook. J. Mater. Res..

[47] Hicks L.D., Dresselhaus M.S. (1993). Effect of Quantum-Well Structures on the Thermoelectric Figure of Merit. Phys. Rev. B.

[48] Terasaki I., Sasago Y., Uchinokura K. (1997). Large Thermoelectric Power in NaCo_2_O_4_ Single Crystals. Phys. Rev..

[49] Shi X., Chen L., Uher C. (2016). Recent Advances in High-Performance Bulk Thermoelectric Materials. Int. Mater. Rev..

[50] Zhao L.D., He J., Berardan D., Lin Y., Li J.F., Nan C.W., Dragoe N. (2014). BiCuSeO Oxyselenides: New Promising Thermoelectric Materials. Energy Environ. Sci..

[51] Zhang X., Chang C., Zhou Y., Zhao L.D. (2017). BiCuSeO Thermoelectrics: An Update on Recent Progress and Perspective. Materials.

[52] Liu P., Chen G., Cui Y., Zhang H., Xiao F., Wang L., Nakano H. (2008). High Temperature Electrical Conductivity and Thermoelectric Power of Na_x_CoO_2_. Solid State Ionics.

[53] Krasutskaya N.S., Klyndyuk A.I., Evseeva L.E., Tanaeva S.A. (2016). Synthesis and Properties of Na_x_CoO_2_ (x = 0.55, 0.89) Oxide Thermoelectrics. Inorg. Mater..

[54] Klyndyuk A.I., Krasutskaya N.S., Chizhova E.A. (2018). Synthesis and Thermoelectric Properties of Ceramics based on Bi_2_Ca_2_Co_1.7_O_y_ Oxide. Glass Phys. Chem..

[55] Sun N., Dong S.T., Zhang B.B., Chen Y.B., Zhou J., Zhang S.T., Gu Z.B., Yao S.H., Chen Y.F. (2013). Intrinsically Modified Thermoelectric Performance of Alkaline-Earth Isovalently Substituted [Bi_2_AE_2_O_4_][CoO_2_]_y_ Single Crystals. J. Appl. Phys..

[56] Chen Y., Chen C., Li X. (2010). Effect on the Properties of Different Preparation Processes in Ca_3_Co_4_O_9_ Thermoelectric Material. Int. Conf. Electr. Control. Eng..

[57] Królicka A.K., Piersa M., Mirowska A., Michalska M. (2018). Effect of Sol-Gel and Solid-State Synthesis Techniques on Structural, Morphological and Thermoelectric Performance of Ca_3_Co_4_O_9_. Ceram. Int..

[58] Noudem J.G., Kenfaui D., Chateigner D., Gomina M. (2012). Toward the Enhancement of Thermoelectric Properties of Lamellar Ca_3_Co_4_O_9_ by Edge-Free Spark Plasma Texturing. Scr. Mater..

[59] Schulz T., Töpfer J. (2016). Thermoelectric Properties of Ca_3_Co_4_O_9_ Ceramics Prepared by an Alternative Pressure-Less Sintering/Annealing Method. J. Alloy. Compd..

[60] Huang Y., Zhao B., Fang J., Ang R., Sun Y. (2011). Tunning of Microstructure and Thermoelectric Properties of Ca_3_Co_4_O_9_ Ceramics by High-Magnetic-Field Sintering. J. Appl. Phys..

[61] Bittner M., Helmich L., Nietschke F., Geppert B., Oeckler O., Feldhoff A. (2017). Porous Ca_3_Co_4_O_9_ with Enhanced Thermoelectric Properties Derived from Sol–Gel Synthesis. J. Eur. Ceram. Soc..

[62] Prasoetsopha N., Pinitsoontorn S., Kamwanna T., Amornkitbamrung V., Kurosaki K., Ohishi Y., Muta H., Yamanaka S. (2014). The Effect of Cr Substitution on the Structure and Properties of Misfit-Layered Ca_3_Co_4−x_Cr_x_O_9+*δ*_ Thermoelectric Oxides. J. Alloy. Compd..

[63] Cha J.S., Choi S., Kim G.H., Kim S., Park K. (2018). High-Temperature Thermoelectric Properties of Sm^3+^-Doped Ca_3_Co_4_O_9+delta_ Fabricated by Spark Plasma Sintering. Ceram. Int..

[64] Wang K.X., Wang J., Wu H., Shaheen N., Zha X.Y., Gao L.J., Bai H.C. (2018). Thermoelectric Properties of Lower Concentration K-Doped Ca_3_Co_4_O_9_ Ceramics. Chin. Phys. B.

[65] Butt S., Xu W., He W.Q., Tan Q., Ren G.K., Lin Y., Nan C.W. (2014). Enhancement of Thermoelectric Performance in Cd-Doped Ca_3_Co_4_O_9_ via Spin Entropy, Defect Chemistry and Phonon Scattering. J. Mater. Chem. A.

[66] Delorme F., Martin C.F., Marudhachalam P., Ovono Ovono D., Guzman G. (2011). Effect of Ca Substitution by Sr on the Thermoelectric Properties of Ca_3_Co_4_O_9_ Ceramics. J. Alloy. Compd..

[67] Hira U., Han L., Norrman K., Christensen D.V., Pryds N., Sher F. (2018). High-Temperature Thermoelectric Properties of Na- and W-Doped Ca_3_Co_4_O_9_ System. RSC Adv..

[68] Butt S., Liu Y.C., Lan J.L., Shehzad K., Zhan B., Lin Y., Nan C.W. (2014). High-Temperature Thermoelectric Properties of La and Fe co-Doped Ca-Co-O Misfit-Layered Cobaltites Consolidated by Spark Plasma Sintering. J. Alloy. Compd..

[69] Ito M., Furumoto D. (2008). Effects of Noble Metal Addition on Microstructure and Thermoelectric Properties of Na_x_Co_2_O_4_. J. Alloy. Compd..

[70] Park K., Choi J.W. (2012). High-Temperature Thermoelectric Properties of Na(Co_0.91_Ni_0.09_)_2_O_4_ Fabricated by Solution Combustion Method for Power Generation. J. Nanosci. Nanotechnol..

[71] Park K., Jang K.U., Kwon H.C., Kim J.G., Cho W.S. (2006). Influence of Partial Substitution of Cu for Co on the Thermoelectric Properties of NaCo_2_O_4_. J. Alloy. Compd..

[72] Nagira T., Ito M., Katsuyama S., Majima K., Nagai H. (2003). Thermoelectric Properties of (Na_1−y_M_y_)_x_Co_2_O_4_(M = K, Sr, Y, Nd, Sm and Yb; Y = 0.01 − 0.35). J. Alloy. Compd..

[73] Karakaya G.Ç., Özçelik B., Nane O., Sotelo A. (2018). Improvement of Bi_2_Sr_2_Co_2_O_y_ Thermoelectric Performances by Na Doping. J. Electroceramics.

[74] Gao F., He Q., Cao R., Wu F., Hu X., Song H. (2015). Enhanced Thermoelectric Properties of the Hole-Doped Bi_2−x_K_x_Sr_2_Co_2_O_y_ Ceramics. Int. J. Mod. Phys. B.

[75] Hao H.S., Ye J.Q., Liu Y.T., Hu X. (2010). High-Temperature Thermoelectric Properties of Pb- and La-Substituted Bi_2_Sr_2_Co_2_O_y_ Misfit Compounds. Adv. Mater. Res..

[76] Janotti A., Van De Walle C.G. (2009). Fundamentals of Zinc Oxide as a Semiconductor. Rep. Prog. Phys..

[77] Van Benthem K., Elsässer C., French R.H. (2001). Bulk Electronic Structure of SrTiO_3_: Experiment and Theory. J. Appl. Phys..

[78] Zhang F.P., Lu Q.M., Zhang X., Zhang J.X. (2011). First Principle Investigation of Electronic Structure of CaMnO_3_ Thermoelectric Compound Oxide. J. Alloy. Compd..

[79] Tsubota T., Ohtaki M., Eguchi K., Arai H. (1997). Thermoelectric Properties of Al-Doped ZnO as a Promising Oxide Material for High-Temperature Thermoelectric Conversion. J. Mater. Chem..

[80] Jood P., Mehta R.J., Zhang Y., Peleckis G., Wang X., Siegel R.W., Borca-Tasciuc T., Dou S.X., Ramanath G. (2011). Al-Doped Zinc Oxide Nanocomposites with Enhanced Thermoelectric Properties. Nano Lett..

[81] Nam W.H., Lim Y.S., Choi S.M., Seo W.S., Lee J.Y. (2012). High-Temperature Charge Transport and Thermoelectric Properties of a Degenerately Al-Doped ZnO Nanocomposite. J. Mater. Chem..

[82] Han L., Van Nong N., Zhang W., Hung L.T., Holgate T., Tashiro K., Ohtaki M., Pryds N., Linderoth S. (2014). Effects of Morphology on the Thermoelectric Properties of Al-Doped ZnO. RSC Adv..

[83] Tian T., Cheng L., Zheng L., Xing J., Gu H., Bernik S., Zeng H., Ruan W., Zhao K., Li G. (2016). Defect Engineering for a Markedly Increased Electrical Conductivity and Power Factor in Doped ZnO Ceramic. Acta Mater..

[84] Ohta S., Nomura T., Ohta H., Koumoto K. (2005). High-Temperature Carrier Transport and Thermoelectric Properties of Heavily La- Or Nb-Doped SrTiO_3_ Single Crystals. J. Appl. Phys..

[85] Han J., Sun Q., Song Y. (2017). Enhanced Thermoelectric Properties of La and Dy co-Doped, Sr-deficient SrTiO_3_ Ceramics. J. Alloy. Compd..

[86] Chen Y., Liu J., Li Y., Zhang X., Wang X., Su W., Li J., Wang C. (2019). Enhancement of Thermoelectric Performance of Sr_1−x_Ti_0.8_Nb_0.2_O_3_ Ceramics by Introducing Sr Vacancies. J. Electron. Mater..

[87] Bittner M., Geppert B., Kanas N., Singh S.P., Wiik K., Feldhoff A. (2016). Oxide-Based Thermoelectric Henerator for High-Temperature Application using p-Type Ca_3_Co_4_O_9_ and n-Type In_1.95_Sn_0.05_O_3_ Legs. Energy Harvest. Syst..

[88] Yan Y.L., Wang Y.X. (2012). Electronic Structure and Low Temperature Thermoelectric Properties of In_24_M_8_O_48_ (M = Ge^4+^, Sn^4+^, Ti^4+^, and Zr^4+^). J. Comput. Chem..

[89] Guilmeau E., Brardan D., Simon C., Maignan A., Raveau B., Ovono Ovono D., Delorme F. (2009). Tuning the Transport and Thermoelectric Properties of In_2_O_3_ Bulk Ceramics through Doping at In-Site. J. Appl. Phys..

[90] Colder H., Guilmeau E., Harnois C., Marinel S., Retoux R., Savary E. (2011). Preparation of Ni-Doped ZnO Ceramics for Thermoelectric Applications. J. Eur. Ceram. Soc..

[91] Zhang D.B., Zhang B.P., Ye D.S., Liu Y.C., Li S. (2016). Enhanced Al/Ni Co-Doping and Power Factor in Textured ZnO Thermoelectric Ceramics Prepared by Hydrothermal Synthesis and Spark Plasma Sintering. J. Alloy. Compd..

[92] Jung K.H., Hyoung Lee K., Seo W.S., Choi S.M. (2012). An Enhancement of a Thermoelectric Power Factor in a Ga-Doped ZnO System: A Chemical Compression by Enlarged Ga Solubility. Appl. Phys. Lett..

[93] Liu J., Wang C.L., Li Y., Su W.B., Zhu Y.H., Li J.C., Mei L.M. (2013). Influence of Rare Earth Doping on Thermoelectric Properties of SrTiO_3_ Ceramics. J. Appl. Phys..

[94] Park K., Son J.S., Woo S.I., Shin K., Oh M.W., Park S.D., Hyeon T. (2014). Colloidal Synthesis and Thermoelectric Properties of La-Doped SrTiO_3_ Nanoparticles. J. Mater. Chem. A.

[95] Li L., Liu Y., Qin X., Li D., Zhang J., Song C., Wang L. (2014). Enhanced Thermoelectric Performance of Highly Dense and Fine-Grained (Sr_1−x_Gd_x_)TiO_3−*δ*_ Ceramics Synthesized by Sol-Gel Process and Spark Plasma Sintering. J. Alloy. Compd..

[96] Zhang B., Wang J., Zou T., Zhang S., Yaer X., Ding N., Liu C., Miao L., Li Y., Wu Y. (2015). High Thermoelectric Performance of Nb-Doped SrTiO_3_ Bulk Materials with Different Doping Levels. J. Mater. Chem. C.

[97] Chen Y., Liu J., Li X., Li Y., Su W., Li J., Zhao L., Wang C., Lu M. (2018). Enhancement of Thermoelectric Performance of Sr_0.9−x_Nd_0.1_Ti_0.9_Nb_0.1_O_3_ Ceramics by Introducing Sr Vacancies. Phys. Status Solidi A.

[98] Bocher L., Aguirre M.H., Logvinovich D., Shkabko A., Robert R., Trottmann M., Weidenkaff A. (2008). CaMn_1−x_Nb_x_O_3_ (x < 0.08) Perovskite-Type Phases As Promising New High-Temperature n-Type Thermoelectric Materials. Inorg. Chem..

[99] Wang Y., Sui Y., Su W. (2008). High Temperature Thermoelectric Characteristics of Ca_0.9_R_0.1_MnO_3_ (R = La,Pr,Yb). J. Appl. Phys..

[100] Thiel P., Eilertsen J., Populoh S., Saucke G., Döbeli M., Shkabko A., Sagarna L., Karvonen L., Weidenkaff A. (2013). Influence of Tungsten Substitution and Oxygen Deficiency on the Thermoelectric Properties of CaMnO_3−*δ*_. J. Appl. Phys..

[101] Seo J.W., Cha J., Won S.O., Park K. (2017). Electrical Transport and Thermoelectric Properties of Ca_0.8_Y_0.2−x_Dy_x_MnO_3−*δ*_ (0 < x < 0.2). J. Am. Ceram. Soc..

[102] Li C., Chen Q., Yan Y. (2018). Effects of Pr and Yb Dual Doping on the Thermoelectric Properties of CaMnO_3_. Materials.

[103] Zhao L.D., Berardan D., Pei Y.L., Byl C., Pinsard-Gaudart L., Dragoe N. (2010). Bi_1−x_Sr_x_CuSeO Oxyselenides as Promising Thermoelectric Materials. Appl. Phys. Lett..

[104] Li F., Li J.F., Zhao L.D., Xiang K., Liu Y., Zhang B.P., Lin Y.H., Nan C.W., Zhu H.M. (2012). Polycrystalline BiCuSeO Oxide as a Potential Thermoelectric Material. Energy Environ. Sci..

[105] Li J., Sui J., Pei Y., Barreteau C., Berardan D., Dragoe N., Cai W., He J., Zhao L.D. (2012). A High Thermoelectric Figure of Merit zT > 1 in Ba Heavily Doped BiCuSeO Oxyselenides. Energy Environ. Sci..

[106] Feng B., Li G., Pan Z., Xiaoming H., Peihai L., Zhu H., Yawei L., Fan X. (2018). Effect of Synthesis Processes on the Thermoelectric Properties of BiCuSeO Oxyselenides. J. Alloy. Compd..

[107] Sui J., Li J., He J., Pei Y.L., Berardan D., Wu H., Dragoe N., Cai W., Zhao L.D. (2013). Texturation Boosts the Thermoelectric Performance of BiCuSeO Oxyselenides. Energy Environ. Sci..

[108] Bhaskar A., Lai R.T., Chang K.C., Liu C.J. (2017). High Thermoelectric Performance of BiCuSeO Prepared by Solid State Reaction and Sol-Gel Process. Scr. Mater..

[109] Pele V., Barreteau C., Berardan D., Zhao L., Dragoe N. (2013). Direct Synthesis of BiCuChO-Type Oxychalcogenides by Mechanical Alloying. J. Solid State Chem..

[110] Stampler E.S., Sheets W.C., Bertoni M.I., Prellier W., Mason T.O., Poeppelmeier K.R. (2008). Temperature Driven Reactant Solubilization Synthesis of BiCuOSe. Inorg. Chem..

[111] Pei Y.L., He J., Li J.F., Li F., Liu Q., Pan W., Barreteau C., Berardan D., Dragoe N., Zhao L.D. (2013). High Thermoelectric Performance of Oxyselenides: Intrinsically Low Thermal Conductivity of Ca-Doped BiCuSeO. NPG Asia Mater..

[112] Li F., Wei T.R., Kang F., Li J.F. (2014). Thermal Stability and Oxidation Resistance of BiCuSeO Based Thermoelectric Ceramics. J. Alloy. Compd..

[113] Lan J.L., Liu Y.C., Zhan B., Lin Y.H., Zhang B., Yuan X., Zhang W., Xu W., Nan C.W. (2013). Enhanced Thermoelectric Properties of Pb-Doped BiCuSeO Ceramics. Adv. Mater..

[114] Li F., Wei T.R., Kang F., Li J.F. (2013). Enhanced Thermoelectric Performance of Ca-Doped BiCuSeO in a Wide Temperature Range. J. Mater. Chem. A.

[115] Sun Y., Zhang C., Cao C., Fu J., Peng L. (2017). Co-Doping for Significantly Improved Thermoelectric Figure of Merit in p-Type Bi_1−2x_Mg_x_Pb_x_CuSeO Oxyselenides. Ceram. Int..

[116] Feng B., Li G., Pan Z., Hou Y., Zhang C., Jiang C., Hu J., Xiang Q., Li Y., He Z. (2018). Effect of Ba and Pb Dual Doping on the Thermoelectric Properties of BiCuSeO Ceramics. Mater. Lett..

[117] Pan L., Lang Y., Zhao L., Berardan D., Amzallag E., Xu C., Gu Y., Chen C., Zhao L.D., Shen X. (2018). Realization of n-Type and Enhanced Thermoelectric Performance of p-Type BiCuSeO by Controlled Iron Incorporation. J. Mater. Chem. A.

[118] Zhang X., Feng D., He J., Zhao L.D. (2018). Attempting to Realize n-Type BiCuSeO. J. Solid State Chem..

[119] Gascoin F., Ottensmann S., Stark D., Haïle S.M., Snyder G.J. (2005). Zintl phases as thermoelectric materials: Tuned transport properties of the compounds CaxYb1-xZn2Sb2. Adv. Funct. Mater..

[120] Gayner C., Kar K.K. (2016). Recent Advances in Thermoelectric Materials. Prog. Mater. Sci..

[121] Brown S.R., Kauzlarich S.M., Gascoin F., Snyder G.J. (2006). Yb_14_MnSb_11_: New High Efficiency Thermoelectric Material for Power Generation. Chem. Mater..

[122] Toberer E.S., Cox C.A., Brown S.R., Ikeda T., May A.F., Kauzlarich S.M., Snyder G.J. (2008). Traversing the Metal-Insulator Transition in a Zintl phase: Rational Enhancement of Thermoelectric Efficiency in Yb_14_Mn_1−x_Al_x_Sb_11_. Adv. Funct. Mater..

[123] Toberer E.S., Brown S.R., Ikeda T., Kauzlarich S.M., Snyder G.J. (2008). High Thermoelectric Efficiency in Lanthanum Doped Yb_14_MnSb_11_. Appl. Phys. Lett..

[124] Cox C.A., Brown S.R., Snyder G.J., Kauzlarich S.M. (2010). Effect of Ca Doping on the Thermoelectric Performance of Yb_14_MnSb_11_. J. Electron. Mater..

[125] Kazem N., Xie W., Ohno S., Zevalkink A., Miller G.J., Snyder G.J., Kauzlarich S.M. (2014). High-Temperature Thermoelectric Properties of the Solid–Solution Zintl Phase Eu_11_Cd_6_Sb_12−x_As_x_ (x < 3). Chem. Mater..

[126] Park S.M., Kim S.J. (2004). Sr_11_Cd_6_Sb_12_: A New Zintl Compound with Infinite Chains of Pentagonal Tubes. J. Solid State Chem..

[127] Kazem N., Hurtado A., Sui F., Ohno S., Zevalkink A., Snyder J.G., Kauzlarich S.M. (2015). High Temperature Thermoelectric Properties of the Solid-Solution Zintl Phase Eu_11_Cd_6−x_Zn_x_Sb_12_. Chem. Mater..

[128] Aydemir U., Zevalkink A., Ormeci A., Wang H., Ohno S., Bux S., Snyder G.J. (2015). Thermoelectric Properties of the Zintl Phases Yb_5_M_2_Sb_6_ (M = Al, Ga, In). Dalton Trans..

[129] Zevalkink A., Swallow J., Snyder G.J. (2013). Thermoelectric Properties of Zn-Doped Ca_5_In_2_Sb_6_. Dalton Trans..

[130] Bobev S., Thompson J.D., Sarrao J.L., Olmstead M.M., Hope H., Kauzlarich S.M. (2004). Probing the Limits of the Zintl Concept: Structure and Bonding in Rare-Earth and Alkaline-Earth Zinc-Antimonides Yb_9_Zn_4+x_Sb_9_ and Ca_9_Zn_4.5_Sb_9_. Inorg. Chem..

[131] Wu Z., Li J., Li X., Zhu M., Wu K.C., Tao X.T., Huang B.B., Xia S.Q. (2016). Tuning the Thermoelectric Properties of Ca_9_Zn_4+x_Sb_9_ by Controlled Doping on the Interstitial Structure. Chem. Mater..

[132] Bux S.K., Zevalkink A., Janka O., Uhl D., Kauzlarich S., Snyder J.G., Fleurial J.P. (2014). Glass-Like Lattice Thermal Conductivity and High Thermoelectric Efficiency in Yb_9_Mn_4.2_Sb_9_. J. Mater. Chem. A.

[133] Kazem N., Zaikina J.V., Ohno S., Snyder G.J., Kauzlarich S.M. (2015). Coinage-Metal-Stuffed Eu_9_Cd_4_Sb_9_: Metallic Compounds with Anomalous Low Thermal Conductivities. Chem. Mater..

[134] Hu Y., Wang J., Kawamura A., Kovnir K., Kauzlarich S.M. (2015). Yb_14_MgSb_11_and Ca_14_MgSb_11_-New Mg-Containing Zintl Compounds and their Structures, Bonding, and Thermoelectric Properties. Chem. Mater..

[135] Tan W., Wu Z., Zhu M., Shen J., Zhu T., Zhao X., Huang B., Tao X.T., Xia S.Q. (2017). A_14_MgBi_11_ (A = Ca, Sr, Eu): Magnesium Bismuth Based Zintl Phases as Potential Thermoelectric Materials. Inorg. Chem..

[136] Toberer E.S., Zevalkink A., Crisosto N., Snyder G.J. (2010). The Zintl Compound Ca_5_Al_2_Sb_6_ for Low-Cost Thermoelectric Power Generation. Adv. Funct. Mater..

[137] Zevalkink A., Toberer E.S., Bleith T., Flage-Larsen E., Snyder G.J. (2011). Improved Carrier Concentration Control in Zn-Doped Ca_5_Al_2_Sb_6_. J. Appl. Phys..

[138] Zevalkink A., Swallow J., Snyder G.J. (2012). Thermoelectric Properties of Mn-Doped Ca_5_Al_2_Sb_6_. J. Electron. Mater..

[139] Chanakian S., Aydemir U., Zevalkink A., Gibbs Z.M., Fleurial J.P., Bux S., Snyder G.J. (2015). High Temperature Thermoelectric Properties of Zn-Doped Eu_5_In_2_Sb_6_. J. Mater. Chem. C.

[140] Lv W., Yang C., Lin J., Hu X., Guo K., Yang X., Luo J., Zhao J.T. (2017). Cd Substitution in Zintl Phase Eu_5_In_2_Sb_6_ Enhancing the Thermoelectric Performance. J. Alloy. Compd..

[141] Chanakian S., Zevalkink A., Aydemir U., Gibbs Z.M., Pomrehn G., Fleurial J.P., Bux S., Snyder G.J. (2015). Enhanced Thermoelectric Properties of Sr_5_In_2_Sb_6_ via Zn-Doping. J. Mater. Chem. A.

[142] Wood M., Aydemir U., Ohno S., Snyder G.J. (2018). Observation of Valence Band Crossing: The Thermoelectric Properties of CaZn_2_Sb_2_-CaMg_2_Sb_2_ Solid Solution. J. Mater. Chem. A.

[143] Zhang H., Baitinger M., Tang M.B., Man Z.Y., Chen H.H., Yang X.X., Liu Y., Chen L., Grin Y., Zhao J.T. (2010). Thermoelectric Properties of Eu(Zn_1−x_Cd_x_)_2_Sb_2_. Dalton Trans..

[144] Guo K., Cao Q.G., Feng X.J., Tang M.B., Chen H.H., Guo X., Chen L., Grin Y., Zhao J.T. (2011). Enhanced Thermoelectric Figure of Merit of Zintl Phase YbCd_2−x_Mn_x_Sb_2_ by Chemical Substitution. Eur. J. Inorg. Chem..

[145] Wang X.J., Tang M.B., Chen H.H., Yang X.X., Zhao J.T., Burkhardt U., Grin Y. (2009). Synthesis and High Thermoelectric Efficiency of Zintl Phase YbCd_2−x_Zn_x_Sb_2_. Appl. Phys. Lett..

[146] Cao Q., Zheng J., Zhang K., Ma G. (2016). Thermoelectric Properties of YbCd_2_Sb_2_ Doped by Mg. J. Alloy. Compd..

[147] Shuai J., Kim H.S., Liu Z., He R., Sui J., Ren Z. (2016). Thermoelectric Properties of Zintl Compound Ca_1−x_Na_x_Mg_2_Bi_1.98_. Appl. Phys. Lett..

[148] Shuai J., Liu Z., Kim H.S., Wang Y., Mao J., He R., Sui J., Ren Z. (2016). Thermoelectric Properties of Bi-Based Zintl Compounds Ca_1−x_Yb_x_Mg_2_Bi_2_. J. Mater. Chem. A.

[149] Shuai J., Geng H., Lan Y., Zhu Z., Wang C., Liu Z., Bao J., Chu C.W., Sui J., Ren Z. (2016). Higher Thermoelectric Performance of Zintl Phases (Eu_0.5_Yb_0.5_)_1−x_Ca_x_Mg_2_Bi_2_ by Band Engineering and Strain Fluctuation. Proc. Natl. Acad. Sci. USA.

[150] Bhardwaj A., Rajput A., Shukla A.K., Pulikkotil J.J., Srivastava A.K., Dhar A., Gupta G., Auluck S., Misra D.K., Budhani R.C. (2013). Mg_3_Sb_2_-Based Zintl Compound: A Non-Toxic, Inexpensive and Abundant Thermoelectric Material for Power Generation. RSC Adv..

[151] Zheng C., Hoffmann R., Nesper R., von Schnering H.G. (1986). Site Preferences and Bond Length Differences in CaAl_2_Si_2_-Type Zintl Compounds. J. Am. Chem. Soc..

[152] Shuai J., Wang Y., Kim H.S., Liu Z., Sun J., Chen S., Sui J., Ren Z. (2015). Thermoelectric Properties of Na-Doped Zintl Compound: Mg_3−x_Na_x_Sb_2_. Acta Mater..

[153] Kim S., Kim C., Hong Y.K., Onimaru T., Suekuni K., Takabatake T., Jung M.H. (2014). Thermoelectric Properties of Mn-Doped Mg-Sb Single Crystals. J. Mater. Chem. A.

[154] Chen X., Wu H., Cui J., Xiao Y., Zhang Y., He J., Chen Y., Cao J., Cai W., Pennycook S.J. (2018). Extraordinary Thermoelectric Performance in n-Type Manganese Doped Mg_3_Sb_2_ Zintl: High Band Degeneracy, Tuned Carrier Scattering Mechanism and Hierarchical Microstructure. Nano Energy.

[155] Wang Y., Zhang X., Wang Y., Liu H., Zhang J. (2019). Enhanced Thermoelectric Properties of n-Type Mg_3_Sb_2_ by Excess Magnesium and Tellurium Doping. Appl. Mater. Sci..

[156] Zhang J., Song L., Pedersen S.H., Yin H., Hung L.T., Brummerstedt Iversen B. (2017). Discovery of High-Performance Low-Cost n-Type Mg_3_Sb_2_-Based Thermoelectric Materials with Multi-Valley Conduction Bands. Nat. Commun..

[157] Shuai J., Ge B., Mao J., Song S., Wang Y., Ren Z. (2018). Significant Role of Mg Stoichiometry in Designing High Thermoelectric Performance for Mg_3_(Sb,Bi)_2_-based n-Type Zintls. J. Am. Chem. Soc..

[158] Shuai J., Mao J., Song S., Zhu Q., Sun J., Wang Y., He R., Zhou J., Chen G., Singh D.J. (2017). Tuning the Carrier Scattering Mechanism to Effectively Improve the Thermoelectric Properties. Energy Environ. Sci..

[159] Graf T., Felser C., Parkin S. (2011). Simple Rules for The Understanding of Heusler Compounds. Prog. Solid State Chem..

[160] Larson P., Mahanti S.D., Sportouch S., Kanatzidis M.G. (1999). Electronic Structure of Rare-Earth Nickel Pnictides: Narrow-Gap Thermoelectric Materials. Phys. Rev. B Condens. Matter Mater. Phys..

[161] Xia Y., Bhattacharya S., Ponnambalam V., Pope A.L., Poon S.J., Tritt T.M. (2000). Thermoelectric Properties of Semimetallic (Zr, Hf)CoSb half-Heusler Phases. J. Appl. Phys..

[162] Bos J.W.G., Downie R.A. (2014). Half-Heusler Thermoelectrics: A Complex Class of Materials. J. Phys. Condens. Matter.

[163] Poon S.J. (2019). Half-Heusler Compounds: Promising Materials For Mid-To-High Temperature Thermoelectric Conversion. J. Phys. D Appl. Phys..

[164] Aliev F.G., Kozyrkov V.V., Moshchalkov V.V., Scolozdra R.V., Durczewski K. (1990). Narrow Band in the Intermetallic Compounds MNiSn (M = Ti, Zr, Hf). Z. Für Phys. B Condens. Matter.

[165] Appel O., Cohen S., Beeri O., Shamir N., Gelbstein Y., Zalkind S. (2018). Surface Oxidation of TiNiSn (half-Heusler) Alloy by Oxygen and Water Vapor. Materials.

[166] Appel O., Breuer G., Cohen S., Beeri O., Kyratsi T., Gelbstein Y., Zalkind S. (2019). The Initial Stage in Oxidation of ZrNiSn (half Heusler) Alloy by Oxygen. Materials.

[167] He R., Kim H.S., Lan Y., Wang D., Chen S., Ren Z. (2015). Investigating the Thermoelectric Properties of p-Type half-Heusler Hf_x_(ZrTi)_1−x_CoSb_0.8_Sn_0.2_ by reducing Hf concentration for power generation. RSC Adv..

[168] Liu Y., Fu C., Xia K., Yu J., Zhao X., Pan H., Felser C., Zhu T. (2018). Lanthanide Contraction as a Design Factor for High-Performance Half-Heusler Thermoelectric Materials. Adv. Mater..

[169] He R., Kraemer D., Mao J., Zeng L., Jie Q., Lan Y., Li C., Shuai J., Kim H.S., Liu Y. (2016). Achieving High Power Factor and Output Power Density in p-Type half-Heuslers Nb_1−x_Ti_x_FeSb. Proc. Natl. Acad. Sci. USA.

[170] Zhu H., He R., Mao J., Zhu Q., Li C., Sun J., Ren W., Wang Y., Liu Z., Tang Z. (2018). Discovery of ZrCoBi Based half-Heuslers with High Thermoelectric Conversion Efficiency. Nat. Commun..

[171] Barth J., Balke B., Fecher G.H., Stryhanyuk H., Gloskovskii A., Naghavi S., Felser C. (2009). Thermoelectric Properties of CoTiSb Based Compounds. J. Phys. D Appl. Phys..

[172] Huang L., Zhang Q., Wang Y., He R., Shuai J., Zhang J., Wang C., Ren Z. (2017). The Effect of Sn Doping on Thermoelectric Performance of n-Type half-Heusler NbCoSb. Phys. Chem. Chem. Phys..

[173] Shutoh N., Sakurada S. (2005). Thermoelectric Properties of the Ti_x_(Zr_0.5_Hf_0.5_)_1−x_NiSn half-Heusler Compounds. J. Alloy. Compd..

[174] Muta H., Kanemitsu T., Kurosaki K., Yamanaka S. (2009). High-Temperature Thermoelectric Properties of Nb-Doped MNiSn (M = Ti, Zr) half-Heusler Compound. J. Alloy. Compd..

[175] Kimura Y., Ueno H., Mishima Y. (2009). Thermoelectric Properties of Directionally Solidified half-Heusler (M_0.5_^a^,M_0.5_^b^)NiSn (M^a^, M^b^ = Hf, Zr, Ti) Alloys. J. Electron. Mater..

[176] Chen L., Gao S., Zeng X., Mehdizadeh Dehkordi A., Tritt T.M., Poon S.J. (2015). Uncovering High Thermoelectric Figure of Merit in (Hf,Zr)NiSn half-Heusler Alloys. Appl. Phys. Lett..

[177] Wu T., Jiang W., Li X., Zhou Y., Chen L. (2007). Thermoelectric Properties of p-Type Fe-Doped TiCoSb half-Heusler Compounds. J. Appl. Phys..

[178] Rowe D. (1995). CRC Handbook of Thermoelectrics.

[179] Lin S., Wang C., Chen H., Huo D., Savvides N., Chen X. (2013). Microstructure and Thermoelectric Properties of Ga-Doped SiGe Alloys Prepared by Mechanical Alloying and Induction Hot Pressing. Funct. Mater. Lett..

[180] Joshi G., Lee H., Lan Y., Wang X., Zhu G., Wang D., Gould R., Cuff D., Tang M., Dresselhaus M. (2008). Enhanced Thermoelectric Figure-of-Merit in Nanostructured p-type Silicon Germanium Bulk Alloys. Nano Lett..

[181] Wang C., Lin S., Chen H., Zhao Y., Zhao L., Wang H., Huo D., Chen X. (2015). Thermoelectric Performance of Si_80_Ge_20−x_Sb_x_ Based Multiphase Alloys with Inhomogeneous Dopant Distribution. Energy Convers. Manag..

[182] Wongprakarn S., Pinitsoontorn S., at Tanusilp S., Kurosaki K. (2018). Enhancing Thermoelectric Properties of p-Type SiGe Alloy through Optimization of Carrier Concentration and Processing Parameters. Mater. Sci. Semicond. Process..

[183] Bathula S., Jayasimhadri M., Singh N., Srivastava A.K., Pulikkotil J., Dhar A., Budhani R.C. (2012). Enhanced Thermoelectric Figure-of-Merit in Spark Plasma Sintered Nanostructured n-Type SiGe Alloys. Appl. Phys. Lett..

[184] Nozariasbmarz A., Agarwal A., Coutant Z.A., Hall M.J., Liu J., Liu R., Malhotra A., Norouzzadeh P., Öztürk M.C., Ramesh V.P. (2017). Thermoelectric Silicides: A Review. Jpn. J. Appl. Phys..

[185] Wang X., Lee H., Lan Y., Zhu G., Joshi G., Wang D., Yang J., Muto A., Tang M., Klatsky J. (2008). Enhanced Thermoelectric Figure-of-Merit in Nanostructured n-Type Silicon Germanium Bulk Alloys. Appl. Phys. Lett..

[186] Zamanipour Z., Vashaee D. (2012). Comparison of Thermoelectric Properties of p-Type Nanostructured Bulk Si_0.8_Ge_0.2_ Alloy with Si_0.8_Ge_0.2_ Composites Embedded with CrSi_2_ Nano-Inclusisons. J. Appl. Phys..

[187] Mackey J., Dynys F., Sehirlioglu A. (2015). Si/Ge–WSi_2_ Composites: Processing and Thermoelectric Properties. Acta Mater..

[188] Usenko A., Moskovskikh D., Korotitskiy A., Gorshenkov M., Voronin A., Arkhipov D., Lyange M., Isachenko G., Khovaylo V. (2016). Thermoelectric Properties of n-Type Si_0,8_Ge_0,2_-FeSi_2_ Multiphase Nanostructures. J. Electron. Mater..

[189] Wongprakarn S., Pinitsoontorn S., Tanusilp S.A., Kurosaki K. (2017). The Effect of YSi_2_ Nanoinclusion on the Thermoelectric Properties of p-Type SiGe Alloy. Phys. Status Solidi (A) Appl. Mater. Sci..

[190] Basu R., Bhattacharya S., Bhatt R., Roy M., Ahmad S., Singh A., Navaneethan M., Hayakawa Y., Aswal D.K., Gupta S.K. (2014). Improved Thermoelectric Performance of Hot Pressed Nanostructured n-Type SiGe Bulk Alloys. J. Mater. Chem. A.

[191] Ahmad S., Singh A., Bohra A., Basu R., Bhattacharya S., Bhatt R., Meshram K.N., Roy M., Sarkar S.K., Hayakawa Y. (2016). Boosting Thermoelectric Performance of p-Type SiGe Alloys through in-situ Metallic YSi_2_ Nanoinclusions. Nano Energy.

[192] Bathula S., Jayasimhadri M., Gahtori B., Kumar A., Srivastava A.K., Dhar A. (2017). Enhancement in Thermoelectric Performance of SiGe Nanoalloys Dispersed with SiC Nanoparticles. Phys. Chem. Chem. Phys..

[193] Nozariasbmarz A., Roy P., Zamanipour Z., Dycus J.H., Cabral M.J., LeBeau J.M., Krasinski J.S., Vashaee D. (2016). Comparison of Thermoelectric Properties of Nanostructured Mg_2_Si, FeSi_2_, SiGe, and Nanocomposites of SiGe-Mg_2_Si, SiGe-FeSi_2_. APL Mater..

[194] Nozariasbmarz A., Zamanipour Z., Norouzzadeh P., Krasinski J.S., Vashaee D. (2016). Enhanced Thermoelectric Performance in a Metal/Semiconductor Nanocomposite of Iron Silicide/Silicon Germanium. RSC Adv..

[195] Zheng G., Su X., Liang T., Lu Q., Yan Y., Uher C., Tang X. (2015). High Thermoelectric Performance of Mechanically Robust n-Type Bi_2_Te_3−x_Se_x_ Prepared by Combustion Synthesis. J. Mater. Chem. A.

[196] Fu C., Zhu T., Liu Y., Xie H., Zhao X. (2015). Band Engineering of High Performance p-Type FeNbSb Based half-Heusler Thermoelectric Materials for Figure of Merit zT > 1. Energy Environ. Sci..

[197] Kleinke H. (2010). New Bulk Materials for Thermoelectric Power Generation: Clathrates and Complex Antimonides. Chem. Mater..

[198] Nolas G.S., Morelli D.T., Tritt T.M. (1999). Skutterudites: A Phonon-Glass-Electron Crystal Approach to Advanced Thermoelectric Energy Conversion Applications. Annu. Rev. Mater. Sci..

[199] Shi X., Yang J., Bai S., Yang J., Wang H., Chi M., Salvador J.R., Zhang W., Chen L., Wong-Ng W. (2010). On the Design of High-Efficiency Thermoelectric Clathrates Through a Systematic Cross-Substitution of Framework Elements. Adv. Funct. Mater..

[200] Zhang L., Grytsiv A., Rogl P., Bauer E., Zehetbauer M. (2009). High Thermoelectric Performance of Riple-Filled n-Type Skutterudites (Sr,Ba,Yb)_y_Co_4_Sb_12_. J. Phys. D Appl. Phys..

[201] Shi X., Yang J., Salvador J.R., Chi M., Cho J., Wang H., Bai S., Yang J., Zhang W., Chen L. (2011). Multiple-Filled Skutterudites: High Thermoelectric Figure of Merit Through Separately Optimizing Electrical and Thermal Transports. J. Am. Chem. Soc..

[202] Zaitsev V.K., Fedorov M.I., Gurieva E.A., Eremin I.S., Konstantinov P.P., Samunin A.Y., Vedernikov M.V. (2006). Highly Effective Mg_2_Si_1−x_Sn_x_ Thermoelectrics. Phys. Rev. B Condens. Matter Mater. Phys..

[203] Khan A.U., Vlachos N.V., Hatzikraniotis E., Polymeris G.S., Lioutas C.B., Stefanaki E.C., Paraskevopoulos K.M., Giapintzakis I., Kyratsi T. (2014). Thermoelectric Properties of Highly Efficient Bi-Doped Mg_2_Si_1−x−y_Sn_x_Ge_y_ Materials. Acta Mater..

